# Human Multi-Lineage Liver Organoid Model Reveals Impairment of CYP3A4 Expression upon Repeated Exposure to Graphene Oxide

**DOI:** 10.3390/cells13181542

**Published:** 2024-09-13

**Authors:** Alessio Romaldini, Raffaele Spanò, Marina Veronesi, Benedetto Grimaldi, Tiziano Bandiera, Stefania Sabella

**Affiliations:** 1 Nanoregulatory Group, D3 PharmaChemistry, Istituto Italiano di Tecnologia, Via Morego 30, 16163 Genoa, Italy; alessio.romaldini@gmail.com (A.R.); raffaele.spano@iit.it (R.S.);; 2 Structural Biophysics Facility, Istituto Italiano di Tecnologia, Via Morego 30, 16163 Genoa, Italy; marina.veronesi@iit.it; 3 D3 PharmaChemistry, Istituto Italiano di Tecnologia, Via Morego 30, 16163 Genoa, Italy; 4 Molecular Medicine, Istituto Italiano di Tecnologia, Via Morego 30, 16163 Genoa, Italy

**Keywords:** 3D cell models, liver organoids, nanomaterials, hepatotoxicity, cytochromes P450, preclinical testing, accumulation, repeated exposure of liver organoid

## Abstract

Three-dimensional hepatic cell cultures can provide an important advancement in the toxicity assessment of nanomaterials with respect to 2D models. Here, we describe liver organoids (LOs) obtained by assembling multiple cell lineages in a fixed ratio 1:1:0.2. These are upcyte^®^ human hepatocytes, UHHs, upcyte^®^ liver sinusoidal endothelial cells, LSECs, and human bone marrow-derived mesenchymal stromal cells, hbmMSCs. The structural and functional analyses indicated that LOs reached size stability upon ca. 10 days of cultivation (organoid maturation), showing a surface area of approximately 10 mm^2^ and the hepatic cellular lineages, UHHs and LSECs, arranged to form both primitive biliary networks and sinusoid structures, alike in vivo. LOs did not show signs of cellular apoptosis, senescence, or alteration of hepatocellular functions (e.g., dis-regulation of CYP3A4 or aberrant production of Albumin) for the entire culture period (19 days since organoid maturation). After that, LOs were repeatedly exposed for 19 days to a single or repeated dose of graphene oxide (GO: 2–40 µg/mL). We observed that the treatment did not induce any macroscopic signs of tissue damage, apoptosis activation, and alteration of cell viability. However, in the repeated dose regimen, we observed a down-regulation of CYP3A4 gene expression. Notably, these findings are in line with recent in vivo data, which report a similar impact on CYP3A4 when mice were repeatedly exposed to GO. Taken together, these findings warn of the potential detrimental effects of GO in real-life exposure (e.g., occupational scenario), where its progressive accumulation is likely expected. More in general, this study highlights that LOs formed by many cell lineages can enable repeated exposure regimens (suitable to mimic accumulation); thus, they can be suitably considered alternative or complementary in vitro systems to animal models.

## 1. Introduction

Hepatotoxicity is one of the major causes of drug failure in clinical trials and drug withdrawal from the market [1,2]. In preclinical assessments, a range of in vitro and in vivo models are usually employed, each having advantages and disadvantages. For example, in the case of in vitro tools, outcomes from microsomes are limited to XME activity, whereas 2D cell models may be limited by poorly differentiated (e.g., immortalized cell lines) or unstable (e.g., primary hepatocytes) phenotype [3,4,5,6]. Also, 2D cultures fail to recapitulate liver cell heterogeneity and architecture and are representative of one or few donors [7,8]. Repeated exposures are challenging due to cellular culture instability, so acute treatments are the dose regimen mostly permitted [8]. On the side of in vivo applications, despite their complexity, animal models better allow the study of drug/nanomaterial organ distribution and clearance; however, they are costly and time-consuming, and outcomes are affected by differences due to interspecies variations with humans, hence often with scarce relevance for screening studies of liver toxicity; finally, higher doses are required in comparison with humans and scarce power to model human population variability and diseases are often reported as the main limitations [7,8,9].

In the framework of preclinical models, advanced 3D liver models are emerging as interesting and alternative tools. For their implementation, generally, a minimal set of parameters should be taken into consideration, such as the incorporation of multiple differentiated cell types, their organization in a 3D structure, and the maintenance of hepatocellular functionality (e.g., expression of phase-I and -II enzymes and transporters, production of Albumin and Urea) for at least 2 weeks [10]. In addition, when human primary cell strains (that can be obtained from healthy or diseased donors) are incorporated, 3D liver models can be potentially even more “human-relevant” tools than animal models to predict human population variability in response to liver metabolism alteration or hepatotoxicity. Hence, they can be considered alternative or complementary systems, contributing to the “3Rs principle” of replacement, reduction, and refinement of animal models [8]. For example, with this regard, Stone and colleagues have constructed models of steatosis and pre-fibrotic non-alcoholic steatohepatitis (NASH), starting from 3D liver models composed of quadruple primary human hepatic cell types, and have used them to assess the hepatotoxicity of a panel of nanomaterials [11]. Liver organoids of different sizes, shapes, and cell compositions have been reported in the literature, ranging from millimeters to a few centimeters [12]. Currently, numerous protocols include parenchymal and non-parenchymal cells from different sources and in different cell amounts to generate in vitro models characterized by an in vivo-like architecture differently from conventional monolayer cultures, thus reproducing cell polarization along with cell–cell and cell–matrix interactions [13,14,15]. The resulting hepatocellular functions have been found stabilized or even improved for extended periods in these models compared to 2D hepatic cell cultures, allowing long-term toxicity investigations [16]. In parallel, the generated microenvironment within the 3D structure generates diffusion gradients of nutrients and oxygen alike tissue in vivo, possibly mimicking access and diffusion of drugs/nanomaterials [15,17]. Therefore, 3D models also offer the possibility to assess drug pharmacokinetics [18]. In this study, we first extensively characterized a trilineage human liver organoid (LO) consisting of upcyte^®^ human hepatocytes (UHHs), upcyte^®^ liver sinusoidal endothelial cells (LSECs), and human bone marrow-derived mesenchymal stromal cells (hbmMSCs), which were seeded on Matrigel^®^-coated 24-well plates according to Ramachandran et al. [19]. In particular, this protocol employs such cell types in a specific ratio of 1:1:0.2 and generates massive 3D aggregates (starting from 1.0 × 10^6^ UHHs, 1.0 × 10^6^ LSECs, and 0.2 × 10^6^ hbmMSCs per single LO) suitable for downstream histological and molecular analyses. However, as an advancement of this study, we cultured LOs for approximately one month with the aim of obtaining a long-term structurally and functionally stable 3D in vitro model. Such a model potentially allows for the testing of toxic substances (such as nanomaterials) in an acute or sub-chronic exposure regimen, according to the indications of OECD Test No. 407, which claims for testing toxicants in an exposure regimen reflecting acute or repeated doses for 28 days [20]. Moreover, we aim to analyze multiple biological endpoints, namely cytotoxicity and hepatocellular functionalities, in the same cell model, as the approach may provide more predictive information on nanomaterial-induced toxicity. To characterize the structural and functional stability of LO over the culture timeframe, we investigated the response to stress signals (e.g., cellular apoptosis and senescence), the acquisition and maintenance of 3D structure (in terms of LO size and spatial organization of UHHs and LSECs), and analyzed the exo-metabolome to indirectly follow some information on energy metabolism during the culture; finally, we followed the functionality of UHHs within LOs. Notably, we employed second-generation UHHs as primary-like hepatocytes in LOs because they may provide some advantages. Being human hepatocytes genetically engineered by upcyte^®^ technology, they can be conditionally expanded in vitro (upon stimulation with OSM) and differentiate into cells with a stable hepatocellular phenotype (after removing OSM) [4]. Furthermore, deriving from human donors, these cells may be representative of population variability and could boost the efficiency of in vitro screening assays. In this study, we employed cells from healthy donors (see Section 2 or see next). We assessed graphene oxide (GO) hepatotoxicity upon exposure of LOs to single or repeated doses. GO was selected as a case study because it is a well-known carbon-based nanomaterial currently tested as a promising material for medical applications. To cite a few, we can refer to the recent use of GO as a carrier of drugs or other bioactive molecules [21] or as a platform for tissue engineering [22], for biosensors [23], and for bioimaging applications [24]. Therefore, LOs were exposed daily to repeated doses of 40 µg/mL GO for up to 19 days (since LO maturation). Specifically, we followed the impact on LO cell viability, with particular attention on apoptosis activation, and the expression of two critical hepatic markers, namely CYP3A4 and Albumin. To the best of our knowledge, only a few data are currently available in the literature, although obtained with 3D spheroids employing murine hepatocytes upon nanomaterial treatment for only 24 h [25]. Specifically, the authors stated that GO treatment reduced the cell viability, altered the tissue histology, and increased ALT and AST levels, showing certain cytotoxicity in spheroids of only murine hepatocytes. Here, our study aims to characterize the GO impact on a human 3D liver model upon repeated exposure (likely mimicking accumulation). The use of LO, which contains multiple human cell types and allows 29 days of culture, can advance the use of 3D human hepatic models in the preclinical assessment of nanomaterial hepatotoxicity.

## 2. Materials and Methods

### 2.1. Materials

UHHs, HHPM with relative supplements, LSECs, and LSEC-specific basal medium with relative supplements were purchased from upcyte^®^ technologies GmbH (Hamburg, Germany). Collagen Type I solution from rat tail, rifampicin, high glucose phenol red-free DMEM, DPBS, resazurin sodium salt, BFC, β-glucuronidase/arylsulfatase, Gill’s No.2 Hematoxylin Solution, and Eosin Y Solution were from Sigma Aldrich (Merck KGaA, Darmstadt, Germany). hbmMSCs and hbmMSC-specific basal medium with relative supplements were from ATCC (Manassas, VA, USA). Cell culture flasks, 96- and 24-well plates, and Corning^®^ Matrigel^®^ basement membrane matrix phenol red free were from Corning Incorporated (Corning, NY, USA). CytoTox96^®^ Non-Radioactive Cytotoxicity Assay was from Promega Corporation (Madison, WI, USA). Human Albumin ELISA Kit was from Bethyl Laboratories, Inc. (Fortis Life Sciences, Boston, MA, USA). Tecan Spark^®^ multimode microplate reader was purchased from Tecan (Männedorf, Switzerland). TRIzol^TM^ Reagent, qPCR primer pairs, Hoechst 33342, and Alexa-Fluor^®^488 Phalloidin were obtained from Invitrogen (Thermo Fisher Scientific, Waltham, MA, USA). NanoDrop One^C^ was from Thermo Fisher Scientific^TM^ (Thermo Fisher Scientific, Waltham, MA, USA). Primary antibody anti-cleaved-PARP was obtained from Thermo Fisher Scientific (Waltham, MA, USA). Primary antibodies anti-CYP3A4, anti-Albumin, anti-GAPDH, anti-cleaved-caspase-3, and anti-VE-cadherin were purchased from Cell Signaling Technology (Danvers, MA, USA). Primary antibody anti-MRP2, secondary Alexa-Fluor^®^555- or Alexa-Fluor^®^488-linked IgG antibodies, and Senescence Detection Kit were from abcam (Cambridge, UK). Primary antibody anti-CD31 was kindly provided by Dr A. Poggi from IRCCS Ospedale Policlinico San Martino (Genoa, Italy). Paraformaldehyde solution was from Santa Cruz Biotechnology Inc. (Heidelberg, Germany). Surgipath^®^ FSC 22 Frozen Section Compound, Leica DM5500 B Microscope, and Leica DMI6000 B Microscope were from Leica Microsystems GmbH (Wetzlar, Germany). Vectashield^®^ Antifade Mounting Medium was obtained from Vector Laboratories (Newark, CA, USA). A1 Confocal Laser Scanning Microscope was purchased from Nikon (Tokyo, Japan). Avance III Ultrashielded Plus 600 MHz FT-NMR Spectrometer, 5-mm NMR tubes, and Assure 4.0 were from Bruker Corporation (Billerica, MA, USA). MestReNova 12.0.3 was from Mestrelab Research (Santiago de Compostela, Spain). Adobe Photoshop was from Adobe (San Jose, CA, USA). GraphPad Prism 6 was from GraphPad Software (San Diego, CA, USA).

### 2.2. Liver Organoid Formation and Culture

Cryopreserved second-generation UHHs (cat. CHE002, donor #653-03) were thawed and expanded for 1 passage in complete HHPM (i.e., fully supplemented Hepatocyte High-Performance Medium) using cell culture flasks coated with 0.1 mL/cm^2^ of 50 μg/mL collagen-type I in 20 mM acetic acid. Similarly, cryopreserved LSECs (cat. CLS002, donor #462) were expanded for 1 passage in complete LSEC culture medium (i.e., fully supplemented LSEC-specific basal medium) using collagen-coated flasks. Cryopreserved hbmMSCs (cat. ATCC-PCS-500-012, lot 63208778) were expanded for more passages in complete hbmMSC culture medium (i.e., fully supplemented hbmMSC-specific basal medium) using un-coated flasks (Supplementary Figure S1A). For the formation of LOs with a 24-well format [19], 1.0 × 10^6^ UHHs, 1.0 × 10^6^ LSECs, and 0.2 × 10^6^ hbmMSCs per LO were mixed, pelleted at 90 g for 5 min, re-suspended in liver-organoid medium (LOM; comprised 50% complete HHPM and 50% complete LSEC culture medium), and seeded in 24-wells coated by 200 μL/cm^2^ of Matrigel^®^ diluted 1:1 (*v*/*v*) with complete LSEC culture medium (referred to as Matrigel^®^-LSECm in Supplementary Figure S1B). LOs were cultured for 29 days, changing the media every day, in incubation at 37 °C in a humidified atmosphere with 5% CO_2_. Photographs of resulting aggregates were taken by a camera at different interval points corresponding to 4 h and days 1, 2, 3, 7, 10, 15, 20, 25, and 29 (Supplementary Figure S1C). In parallel, UHHs were cultured in collagen-coated 12-wells (1.0 × 10^6^ cells/well) with LOM for 10 days, changing the media every day, in incubation at 37 °C in a humidified atmosphere with 5% CO_2_. Each day, the LO- or UHH-conditioned media were collected, clarified at 15,000× *g* for 15 min at 4 °C, and frozen only once at −20 °C for downstream investigations.

### 2.3. Cytotoxicity Assay

The CytoTox96^®^ Non-Radioactive Cytotoxicity Assay was used to estimate the cell membrane integrity during the LOs self-assembling. On days 1, 2, 3, 7, 10, 15, 20, 25, and 29, the LO-conditioned media were daily saved and conserved as described above. In parallel to LO culture, LOM was incubated in LO-free, Matrigel^®^-coated wells under the same culture conditions, processed as described for LO-conditioned media, and used as the blank value to subtract during the data analysis. Samples were assessed following the manufacturer’s instructions. Results are expressed as mean ± SD of eight independent LOs per time point.

### 2.4. NMR-Based Exo-Metabolomics

The NMR-based exo-metabolomics was performed on LO-conditioned media collected on days 1, 2, 3, 7, 10, 15, 20, 25, and 29, and on UHH-conditioned media collected on days 1, 2, 3, 7, and 10, and immediately frozen and stored at −80 °C. The day of the analysis, the LO- or UHH-conditioned media were thawed on ice and pelleted at 13,000 g for 5 min at 4 °C. For each supernatant, 400 µL were transferred into 5 mm NMR tubes and 100 µL of a solution comprised 750 mM phosphate buffer at pH 7.4, 5 mM TSP, and 0.2% NaN3 in D_2_O (final concentration: 150 mM phosphate buffer at pH 7.4, 1 mM TSP, 0.04% NaN3, and 20% D_2_O) were added. TSP was used as the reference compound for the ^1^H chemicals shift and D_2_O for the lock signal. All the spectra were recorded at 298 K on an Avance III and Avance NEO Ultrashielded Plus 600 MHz FT-NMR Spectrometer, equipped with a CryoProbeTM QCI ^1^H/^19^F-^13^C/^15^N-D and a SampleJetTM autosampler with temperature control. The tubes were conditioned for 3 min at the experimental temperature before their insertion into the magnet; then, the probe was automatically locked, tuned, matched, shimmed, and the samples’ temperature stability was checked for a further 3 min before the acquisition. For each sample, a 1D ^1^H-NMR spectrum was recorded with the standard NOESY (nuclear Overhauser effect spectroscopy) presat pulse sequence for the water suppression (64 k data points, a spectral width of 29.75 ppm, 128 scans, an acquisition time of 1.83 s, a relaxation delay (d1) of 4 s and a mixing time of 100 μs) and a 1D ^1^H spin-echo Carr-Purcell-Meiboom-Gill pulse sequence (Meiboom and Gill, 1958 [26]) for the suppression of NMR signals from large macromolecules, such as proteins and other substances with short T_2_ values (total echo time to 80 ms, 128 scans, 64 k data points, an acquisition time of 1.83 s, and a d1 of 4 s). All the spectra were Fourier transformed and phase corrected, applying a line broadening of 0.3 Hz, a zerofilling to 128 k points, and were baseline corrected by applying a polynomial baseline correction.

The Principal Component Analysis (PCA) was run on MestReNova 12.0.3 using a bin with 0.04 ppm for the bucketing, normalizing the data by the sum of all signals, and scaling using Pareto and a number of components of seven. The identification of the exo-metabolites and the relative quantification were obtained using Assure 4.0 and BBIOREFCODE database, both from Bruker. At each time interval, the consumption or secretion of each exo-metabolite in the LO- or UHH-conditioned media was calculated considering the relative basal level found in LOM incubated in LO- or UHH-free, Matrigel^®^- or collagen-coated wells under the same culture conditions, respectively. Results are expressed as mean ± SD of eight independent LOs or four UHH-based replicates per time point.

### 2.5. LO 2D Projection Area Measurements

Self-assembling and development of LOs were followed over time by measuring the total surface area occupied by each LO in the well. On days 1, 2, 3, 7, 10, 15, 20, 25, and 29, multiple brightfield images per LO were acquired with a Leica DMI6000 B Microscope (5× magnification; see Step 0 in Supplementary Figure S1D) and used to generate a stitched image of the complete 2D projection of that sample through the automatic tool “Photomerge” (File→Automate→Photomerge→Reposition option) of Adobe Photoshop (see Step 1 in Supplementary Figure S1D). Fiji [27] was used to measure the total area of each LO 2D projection, using the “Polygon selection” tool to create an ROI along the LO perimeter and to obtain the selection area, finally reported in mm^2^ (see Step 2 in Supplementary Figure S1D). The schematic of the entire process is shown in Supplementary Figure S1D (Steps 1–3). Results are expressed as mean ± SD of eight independent LOs per each time point.

### 2.6. CYP3A4 Activity Assay

The metabolic activity of CYP3A4 was measured in LOs cultured for 10 or 29 days by incubation of 7-benzyloxy-4-trifluoromethylcoumarin (BFC), as described in Romaldini et al. with some modifications [28]. At the end of any time interval, LOs were washed three times with DPBS (1 mL/24-well), removed from Matrigel^®^, transferred into new 24-well plates, and further washed with DPBS. For each LO, 300 μL of 100 μM BFC in the incubation medium (1 mM Na_2_HPO_4_, 137 mM NaCl, 5 mM KCl, 0.5 mM MgCl_2_, 2 mM CaCl_2_, 10 mM glucose, 10 mM Hepes; pH 7.4 buffered solution) were added, incubated for 4 h at 37 °C in a humidified atmosphere with 5% CO_2_, transferred into a new 96-well plate (50 μL per well), diluted 1:1 (*v*/*v*) with β-glucuronidase/arylsulfatase (approximately 150 Fishman units/mL and 1200 Roy units/mL, respectively), and further incubated for 4 h at 37 °C. The reaction was stopped by diluting 1:1 (*v*/*v*) such mixture with the quenching solution (0.25 M Tris in 60% acetonitrile). The 7-hydroxy-4-trifluoromethylcoumarin (HFC) formation was quantified at 410 nm (excitation) and 510 nm (emission) by a Tecan Spark^®^ reader. This procedure was repeated by adding the same reagents into empty wells to have blank values to subtract during the data analysis. Results are expressed as mean ± SD of three independent LOs per time point.

### 2.7. Albumin Quantification

The concentration of secreted Albumin was measured in LO-conditioned media saved on days 10 and 29 by Human Albumin ELISA Kit. The LO-conditioned media were daily saved and conserved as described above. In parallel to LO culture, LOM was incubated in LO-free, Matrigel^®^-coated wells under the same culture conditions, processed as described for LO-conditioned media, and used as the blank value to subtract during the data analysis. Samples were assessed following the manufacturer’s instructions. Results are expressed as mean ± SD of three independent LOs per time point.

### 2.8. Liver Organoid Treatment

LOs cultured for 10 days were treated with 40 µg/mL GO or less (final volume, 1 mL per well; well area, approximately 2.0 cm^2^) for up to 19 days, using daily repeated doses. After 24 h of incubation at 37 °C in a humidified atmosphere with 5% CO_2_, the GO-supplemented LOM was removed from each well and replaced with a freshly prepared stimulation medium after washing with DPBS. To induce CYP3A4 gene expression, LOs cultured for 7–10 days were treated with 100 µM Rifampicin for 3 days, changing the stimulation medium every day [29]. The same protocols were followed in parallel for the control LOs, using un-supplemented LOM.

### 2.9. Graphene Oxide Characterization

Graphene oxide (GO) was kindly provided by Dr E. Vazquez from Universidad de Castilla-La Mancha (Spain). Our group has already reported an exhaustive physico-chemical characterization of the same nanomaterial. In particular, GO has the typical flake-like shape, as revealed by TEM and SEM investigations, is characterized by a high content of oxygen atoms, as demonstrated by EDS and thermogravimetric analysis, and exhibits a broad size distribution with the most represented size of 300 nm, as obtained by DLS and TEM measurements [28,30,31]. Considering that LOs were exposed to daily repeated GO doses for up to 19 days, the colloidal stability of 40 µg/mL GO in LOM was evaluated over 24 h by DLS analysis as described in Romaldini et al. [28]. After 0, 2, and 24 h of incubation in LOM, GO was pelleted at 15,000× *g* for 15 min at 4 °C, washed three times with Milli-Q^®^ water adding a volume equal to the suspension volume, and characterized by Zeta Potential analysis as previously reported [28].

### 2.10. Cell Viability Assay

The resazurin reduction assay was used to measure the cell viability of LOs cultured for 10 or 29 days, and LOs treated or not with GO for 1 or 19 days. At the end of any time interval, LOs were washed three times with DPBS (1 mL/24-well), removed from Matrigel^®^, transferred into new 24-well plates, and further washed with DPBS. For each LO, 300 μL of serum-free phenol red-free high glucose DMEM, supplemented with 44 μM resazurin sodium salt [32], were added, incubated for 1 h at 37 °C in a humidified atmosphere with 5% CO_2_ in the dark, and transferred into a new 96-well plate (100 μL per well). Fluorescence was measured at 535 nm by a Tecan Spark^®^ reader. In parallel, the resazurin solution was also added into empty 24-wells, incubated under the same conditions, and used as the blank value to subtract during the data analysis. Results are expressed as percentage values over the mean value (set as 100%) relative to the control condition (i.e., day 10 or un-treated) and represent mean ± SD of two to four independent LOs per experimental condition.

### 2.11. Gene Expression Analysis

The gene expression analysis was carried out on LOs cultured for 10 or 29 days, treated or not with GO for 1 or 19 days, and treated or not with rifampicin for 3 days. At the end of any incubation, LOs were washed three times with DPBS (1 mL/24-well), removed from Matrigel^®^, transferred into new 24-well plates, and further washed with DPBS. For each LO, 500 μL of TRIzol^TM^ Reagent were added and incubated at −80 °C for at least one night. Total RNA was isolated from each sample, reverse transcribed to first-strand cDNA, and analyzed by quantitative Real Time PCR (qPCR) as described in Romaldini et al. [28]. Primer sequences and relative technical details are listed in Supplementary Table S1. The relative gene expression of a target gene was calculated using Pfaffl’s model [33]. GAPDH was selected as the reference gene for data normalization. Each reaction was performed in technical triplicate. Unless noted otherwise, results are expressed as mean ± SD of three independent LOs per experimental condition.

### 2.12. DNA Quantification

The DNA content was isolated from LOs cultured for 10 or 29 days using the same homogenates generated for RNA isolation as described by Chomczynski [34]. Each sample was quantified by NanoDrop One^C^. Results are expressed as mean ± SD of three independent LOs per time point.

### 2.13. Western Blot

Proteins were isolated from LOs cultured for 10 or 29 days and treated or not with GO for 1 or 19 days, quantified, and separated as described in Romaldini et al. [28]. Target proteins were detected using specific primary antibodies (cleaved PARP, 1:1000 *v*/*v*; CYP3A4, 1:1000 *v*/*v*; Albumin, 1:1000 *v*/*v*) as previously reported [28]. In parallel with the gene expression analysis, GAPDH was used as the internal control (primary antibody diluted 1:1000 *v*/*v*). In the densitometric analysis, Fiji [27] was used to quantify band densities. Results are reported as the n-fold increase over the mean value relative to the control condition (set as 1.0). Unless noted otherwise, results are expressed as mean ± SD of three independent LOs per experimental condition.

### 2.14. Histology

Histological analysis was performed on LOs cultured for 10 or 29 days and treated or not with GO for 1 or 19 days. At the end of any incubation, LOs were washed three times with DPBS (1 mL/24-well), removed from Matrigel^®^, transferred into new 24-well plates, and further washed with DPBS. Each LO was transferred into a 1.5 mL tube and fixed with 4% paraformaldehyde solution in DPBS at room temperature for 1 h on a rotating mixer. At the end of that incubation, samples were washed three times with DPBS (1 mL/tube) and prepared for cryo-histology using an upgraded sucrose solution series (10%, 20%, and 30% sucrose in DPBS). Each sample was kept in 10% or 20% sucrose solution for 1 h at room temperature, whereas for at least 24 h at 4 °C in the 30% sucrose solution. After removing the sucrose solution, tubes were put onto an opened Petri dish floating on liquid nitrogen for a few minutes to quickly freeze the sample and conserved at −80 °C. Each sample was embedded in FSC 22 Frozen Section Compound at a working temperature of −20 °C and cut into 10 μm-thin sections.

For Hematoxylin and Eosin (H&E) staining, sections were stained with Gill’s No.2 Hematoxylin Solution for 2 min, counterstained with Eosin Y Solution for 1 min, dehydrated in upgraded ethanol, cleared with xylene, and mounted in Permount^TM^ Mounting Medium. Images were captured by a Leica DM5500 B Microscope. For immunofluorescence staining, sections were permeabilized with 0.1% Triton X-100 in DPBS for 10 min at room temperature, blocked with 3% BSA in 0.1% Triton X-100 in DPBS for 1 h at room temperature, incubated for 2 h at room temperature in a humid chamber with a specific primary antibody diluted in 1% BSA in DPBS (cleaved caspase-3, 1:400 *v*/*v*; MRP2, 1:100 *v*/*v*; CD31, undiluted; VE-cadherin 1:400 *v*/*v*). To visualize the primary antibody, sections were incubated for 1 h at room temperature in a humid chamber in the dark with secondary anti-rabbit or anti-mouse, Alexa-Fluor^®^555- or Alexa-Fluor^®^488-linked IgG antibodies, diluted 1:200 (*v*/*v*) in 1% BSA in DPBS. For nuclear counterstaining, sections were incubated for 30 min at room temperature with Hoechst 33342 diluted 1:1000 (*v*/*v*) in 1% BSA in DPBS. To selectively stain the F-actin cytoskeleton, sections were incubated for 30 min at room temperature with Alexa-Fluor^®^488 Phalloidin diluted 1:50 (*v*/*v*) in 1% BSA in DPBS. Finally, sections were mounted with Vectashield^®^ Antifade Mounting Medium. Images were captured by A1 Confocal Laser Scanning Microscope.

### 2.15. SA-β-gal Activity Detection

The Senescence Detection Kit was used for the histochemical detection of senescence-associated β-galactosidase (SA-β-gal) activity in frozen sections of LOs cultured for 10 or 29 days. Sections were incubated with the staining solution mix at 37 °C overnight, washed with Milli-Q^®^ water, and mounted with Vectashield^®^ Antifade Mounting Medium. Images were captured by a Leica DM5500 B Microscope. For quantifying SA-β-gal^+^ cells, six to eight identical regions of interest were selected in 10× images, and positive cells were counted. For each sample, the number of positive cells per mm^2^ is reported. Results are expressed as mean ± SD relative to at least three independent LOs per time point.

### 2.16. Statistical Analysis

Statistical analysis was run on GraphPad Prism 6. A confidence interval of 95% was set in each test performed. When *p* < 0.05 was obtained, the analyzed difference was considered statistically significant. Ordinary one-way ANOVA was used for the LO 2D projection area measurements, cytotoxicity assay, NMR-based exo-metabolomics, Western blot analysis on LOs treated or not with GO for 1 day, and cell viability assay on LOs treated or not with GO for 1 day. If ANOVA found statistically significant differences, Tukey’s multiple comparisons test was used for the post hoc analysis. Two-way ANOVA with Sidak’s multiple comparisons test was used for the gene expression analysis. Unpaired Mann–Whitney U test was used for the cell viability assay on LOs cultured for 10 or 29 days and treated or not with GO for 29 days, Western blot analysis on LOs cultured for 10 or 29 days and treated or not with GO for 29 days, CYP3A4 activity assay, DNA quantification, SA-β-gal activity detection, and gene expression analysis on LOs cultured for 10 or 29 days (only for PUMA and CDKN1A). Unless noted otherwise, results are expressed as mean ± SD of three independent LOs per experimental condition.

## 3. Results and Discussion

### 3.1. Structural and Functional Characterization of LOs

#### 3.1.1. Self-Assembling of LOs and Characterization of Their Size Stability

**Figure 1:**
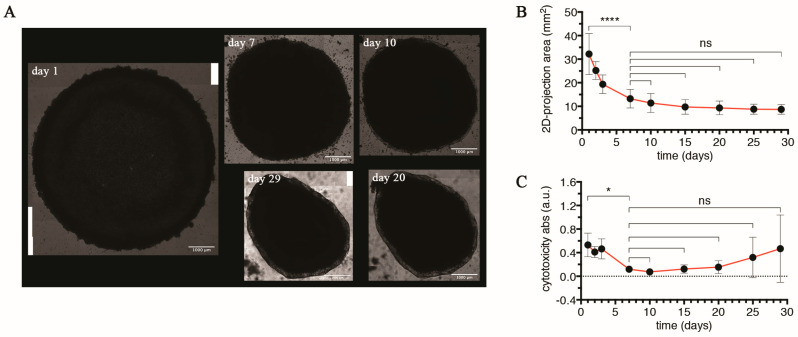
Self-assembling of three cell lineages (UHHs, LSECs, and hbmMSCs) mixed in a ratio of 1:1:0.2 and size stability of LO over time. (**A**) Reconstruction of representative images of LO on days 1, 7, 10, 20, and 29 (scale bar = 1000 μm). (**B**) Two-dimensional projections of surface area of LO at defined time intervals (day 1, 2, 3, 7, 10, 15, 20, 25, and 29) measured by Adobe Photoshop and Fiji (further information in Section 2). For each time point, results are expressed as mean ± SD of eight independent measurements. The symbols ‘****’ and ‘ns’ refer to *p* < 0.0001 and *p* > 0.05, respectively (ordinary one-way ANOVA). (**C**) Cell membrane integrity damage over time by cytotoxicity assay (LDH assay). Results represent mean ± SD of eight independent measurements per time point. The symbols ‘*’ and ‘ns’ refer to *p* = 0.0342 and *p* > 0.05, respectively (ordinary one-way ANOVA).

In 2013, Takebe et al. demonstrated, for the first time, that in vitro human liver buds were able to generate vascularized and functional human liver after ectopic transplantation into mice [35]. Specifically, such liver buds originated by self-assembling of three cell lineages, namely human-induced pluripotent stem cell-derived hepatic endodermal cells, human umbilical vein endothelial cells, and MSCs mixed in an appropriate ratio. When plated on Matrigel^®^, such mixed populations started to arrange to form 3D aggregates with MSCs essential for their formation. Following such evidence, we prepared liver organoids (LOs) mixing three primary human cell types, such as UHHs, LSECs, and hbmMSCs, in a ratio of 1:1:0.2, respectively (Supplementary Figure S1A), according to the protocol developed by Ramachandran et al. [19]. LOs were cultured into Matrigel^®^-coated 24-well plates (one LO per well) under static conditions for 29 days (Supplementary Figure S1B,C). Initially, we observed that the cells seeded on the Matrigel^®^ formed a 2D cell layer, and over time, this 2D structure reorganized into a 3D architecture. The clear generation of LOs was observed within the 4–24 h timeframe, as shown in the multi-panel image (Supplementary Figure S1C). During this period, the cells that were initially spread around the well started to rearrange into a more close-knit structure. This temporal window of formation is in line with the observation reported by Ramachandran et al. [19]. Figure 1A shows a representative mature LO, which appears as a compact, massive 3D cellular aggregate. We provided a size characterization of LOs over time. During the first 7 days, we observed that the size of LOs (expressed as 2D projections area) gradually decreased to a value of approximately 13.2 mm^2^ compared to day 1 (32.2 mm^2^; *p* < 0.0001; Figure 1B); after that, it remained relatively constant until the end of culture (from day 7 to day 29), suggesting that LOs reached size stability and maturation. To investigate the impact of the self-assembling process to cells, we measured the level of the cytosolic enzyme lactate dehydrogenase (LDH) released into the LO-conditioned medium as an indicator of cell membrane integrity damage. Figure 1C indicated that LDH levels were relatively constant in the first three days but decreased on day 7 (*p* = 0.0342) and remained unvaried until day 20. In the last two time points (days 25 and 29), we found a gradual increase in LDH release, even though it was not significant due to a high standard deviation. The observed trend suggests that cells within LO achieved a good health status when assembled in a 3D structure (from day 7), whereas, at longer times (from day 25), they seemed likely to experience more stressful conditions.

#### 3.1.2. Exo-Metabolome of LOs by NMR: Aerobic Glycolysis Supports LO Growth and Stabilization

The generation of spatially organized 3D cellular aggregates by chaotic 2D cell mixtures required specific bio-energetic demands due to local cellular rearrangements, thus cell metabolism was expected to change during the assembling process [36]. Among omics techniques, metabolomics, including ^1^H NMR exo-metabolomics, by disclosing changes in metabolites in cells, tissues, and bio-fluids, can provide a snapshot of the functional and physiological status of a system providing a whole picture of metabolic active molecule products; it definitely provides a downstream picture reflecting all changes occurring at the genetic, transcript, and protein level, which, however, occur at different cellular expression timeframes; therefore, it is considered the technique, among the other omics, which provides information of the organism closest to the phenotype [37,38,39]. ^1^H NMR exo-metabolomics has been reported to be a rapid, effective, and reliable approach for monitoring cell metabolism [40]. In general, it quantifies metabolites consumed from or secreted into the growth medium, providing specific metabolic footprinting [40,41,42,43]. Recently, ^1^H NMR exo-metabolomics has been used to evaluate the possible toxic effects of drugs, nano- and smart materials [44,45,46].

**Figure 2:**
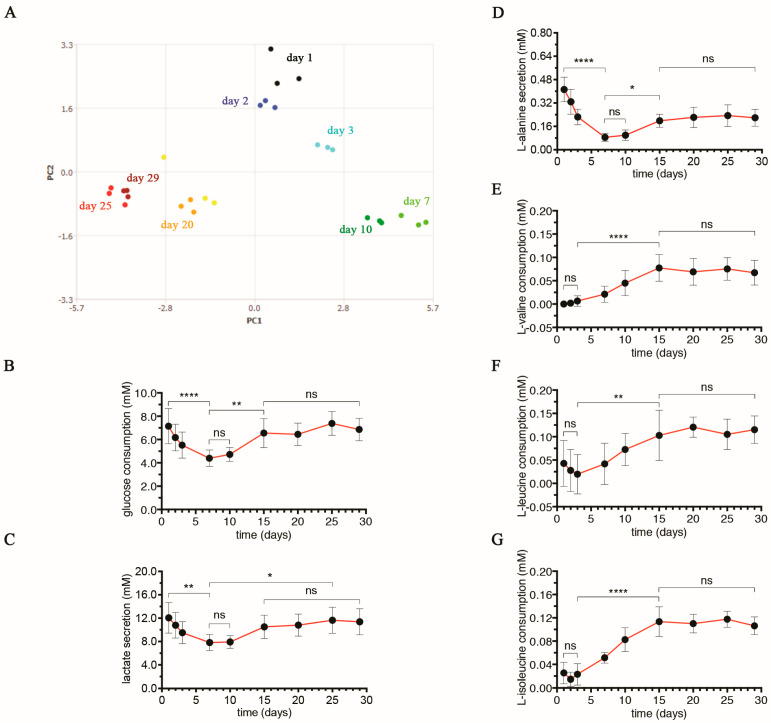
Characterization of the energy metabolism during LO culture. (**A**) Representative PCA performed on the 1D ^1^H-NMR spectra (obtained by one of three experimental set-ups used) relative to 3 LO-conditioned media at defined time intervals (day 1, 2, 3, 7, 10, 15, 20, 25, and 29). Comparable results were found running the PCA on 1D ^1^H-NMR spectra relative to other two experimental set-ups (not included). (**B**,**C**) Consumption of glucose (**B**) and secretion of lactate (**C**) by LOs over time, calculated considering the relative basal level found in LOM incubated in cell-free, Matrigel^®^-coated wells under the same culture conditions. For each time point, results are expressed as mean ± SD of eight independent LOs. The symbols ‘*’, ‘**’, ‘****’, and ‘ns’ refer to *p* = 0.0124, *p* ≤ 0.0042, *p* < 0.0001, and *p* > 0.05, respectively (ordinary one-way ANOVA). (**D**) Secretion of L-alanine by LOs over time, calculated considering the relative basal level found in LOM incubated in cell-free, Matrigel^®^-coated wells under the same culture conditions. For each time point, results are expressed as mean ± SD of eight independent LOs. The symbols ‘*’, ‘****’, and ‘ns’ refer to *p* = 0.0137, *p* < 0.0001, and *p* > 0.05, respectively (ordinary one-way ANOVA). (**E**–**G**) Consumption of L-valine (**E**), L-leucine (**F**), and L-isoleucine (**G**) by LOs over time, calculated considering the relative basal level found in LOM incubated in cell-free, Matrigel^®^-coated wells under the same culture conditions. For each time point, results represent means ± SD of eight independent LOs. The symbols ‘**’, ‘****’, and ‘ns’ refer to *p* = 0.0051, *p* < 0.0001, and *p* > 0.05, respectively (ordinary one-way ANOVA).

Therefore, we investigated the extracellular molecular content of LO-conditioned media over the entire culture period (referred to as exo-metabolome in the text) to highlight the metabolic efforts of the cell types during organoid formation. 1D ^1^H-NMR spectra were acquired for LO-conditioned media at different culturing times (i.e., 1, 2, 3, 7, 10, 15, 20, 25, and 29 days; Figure 2A). The Principal Component Analysis (PCA) was performed on the spectra related to LO-conditioned media using MestReNova 12.0.3. The obtained results highlighted three groups with different metabolic behavior. Indeed, it shows a clear separation along the two principal components (PC1 and PC2) for samples cultured at days 1–3 from those cultured at days 15–29, suggesting a strong dissimilarity in the molecular medium content. Interestingly, for samples relative to days 7 and 10, PCA highlights an intermediate situation. This trend suggests that the cellular metabolism changes during organoid maturation (until day 7–10; see Figure 1A) and continues to gain relevant changes up to day 15, when cells appear to acquire certain metabolic stability.

Using both the Bruker Assure 4.0 program and the BBIOREFCODE database, we were able to identify and quantify about 20 metabolites whose content varied during the culture. Their consumption or secretion was quantified by comparing their amounts between LO-conditioned media and control medium (i.e., liver-organoid medium without LO) at the corresponding culture times. Six metabolites (glucose, lactate, L-alanine, L-valine, L-leucine, and L-isoleucine; Figure 2B–G) showed significant differences (by one-way ANOVA with Bonferroni correction and 95% confidence intervals). In particular, we found that the glucose consumption significantly decreased in the first 7 days (*p* < 0.0001 for day 1 vs. day 7), was relatively stable until day 10, slightly increased on day 15 (*p* < 0.0042 for day 7 vs. day 15), and remained unchanged until the end of the culture time (Figure 2B). Park et al. demonstrated that the glycolysis rate is influenced by mechanical stimuli (e.g., stiffness) of the local microenvironment and declines when untransformed cells grow on soft substrates compared to stiffer substrates [47]. In our model, cells first experience the Matrigel^®^-coated plastic wells and subsequently the extracellular matrix produced during the rearrangement of cells from 2D to 3D assembly; therefore, adjusting the glycolysis rate in response to environmental mechanics is plausible. Indeed, we observed that glycolysis slowed down from day 1 to day 7, when the most dramatic phase of spatial cellular rearrangement occurred, whereas it arrived at constant glucose consumption from day 15 to day 29, when LOs were already formed and dimensionally stable. In parallel, the secretion of lactate followed a trend almost superimposable to glucose consumption, further indicating a modulated glycolytic rate over time (Figure 2C). In particular, the lactate to glucose molar ratio ranged from 1.6 to 1.8 with no statistical differences among the time intervals considered (Supplementary Figure S2A), suggesting greater use of aerobic glycolysis (ratio commonly close to 2) than oxidative phosphorylation (OXPHOS; ratio much lower than 2) to generate energy over the entire culture even in the presence of oxygen (a phenomenon called the Warburg effect) [48]. Indeed, the conversion of glucose to lactate is a less efficient way to generate energy in terms of adenosine 5′-triphosphate (ATP) production (2 moles of ATP per mole of glucose) compared to OXPHOS (36 moles of ATP per mole of glucose). When resources are abundant, or the oxygen supply is inadequate, glycolysis is more convenient due to a higher rate of ATP production [49]. In addition, this energetic pathway guarantees not only free energy but also biomass and, thus, could meet the metabolic requirements of a complex system in growth [50]. Such a preferential use of aerobic glycolysis by LOs seems to be an intrinsic feature of LO-forming cells rather than merely a metabolic reprogramming in response to external cues (e.g., stiffness of the extracellular microenvironment or oxygen content within the tissue). In support of our hypothesis, UHHs (in our cell model, about 45.5% of the total cell population) cultured in a 2D format for 10 days under conditions similar to those used for LOs (i.e., 1.0 × 10^6^ cells in liver-organoid medium, LOM) comparably showed a lactate to glucose molar ratio ranging from 1.5 to 1.6 starting from day 2 (Supplementary Figure S2B), thus indicating that the energetic pathway chosen by UHHs is possibly independent of the culture system format (2D or 3D). Similarly, it is reported in the literature that endothelial cells (in our cell model, represented by LSECs as about 45.5% of the total cell population) also physiologically prefer aerobic glycolysis to OXPHOS, even in the presence of adequate amounts of oxygen guaranteed by their location close to the bloodstream [51].

The metabolic stability showed by LOs after 15 days of culture is also indirectly confirmed by the secretion of L-alanine, which was relatively constant between days 15 and 29 (Figure 2D). Interestingly, we also found that the consumption of branched-chain amino acids (BCAA) L-valine, L-leucine, and L-isoleucine was almost constant in the first three days and then significantly increased, reaching a plateau on day 15 (*p* < 0.0001, *p* = 0.0051, and *p* < 0.0001, respectively, for day 3 vs. day 15) until day 29 (Figure 2E–G). BCAA catabolism provides acetyl-CoA and succinyl-CoA [52], which can enter the tricarboxylic acid (TCA) cycle among many other metabolic pathways and, thus, can contribute to generating ATP via OXPHOS or glucose via gluconeogenesis [53,54].

Taken together, these data suggest LOs reached a steady state in metabolism on day 15, which is described here by aerobic glycolysis and BCAA catabolism with no further appreciable variations until the culture end.

#### 3.1.3. LOs Do Not Exhibit Signs of Apoptosis or Cellular Senescence until Day 29

**Figure 3:**
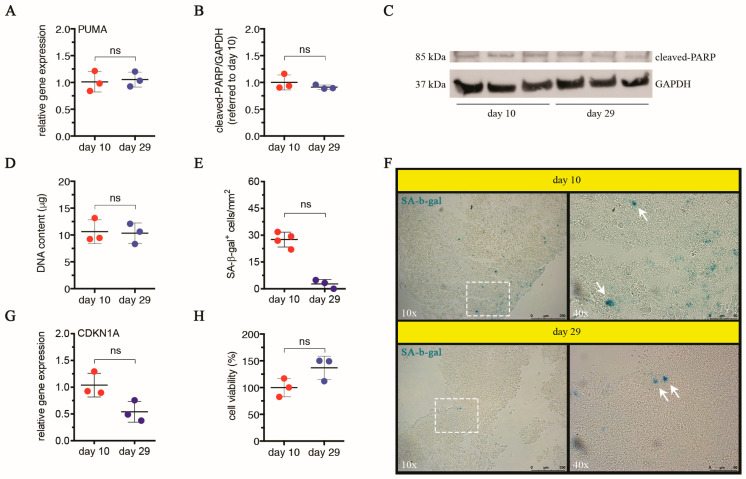
Apoptosis and cellular senescence within LOs over time. (**A**) Relative gene expression of PUMA in LOs cultured for 29 days compared to LOs cultured for 10 days, analyzed by qPCR. Results are expressed as mean ± SD of three independent LOs per time point. The symbol ‘ns’ refers to *p* > 0.05 (unpaired Mann–Whitney U test). (**B**,**C**) Cleaved-PARP levels in LOs cultured for 29 days compared to LO, cultured for 10 days, analyzed by Western blot. Densitometry analysis of band intensities relative to cleaved PARP (**B**) and the corresponding blot relative to three independent LOs per time point, probed with primary antibodies anti-cleaved PARP and anti-GAPDH, are shown (**C**). GAPDH was used as internal control. Results are expressed as n-fold increase over the mean value relative to day 10. The symbol ‘ns’ refers to *p* > 0.05 (unpaired Mann–Whitney U test). (**D**) Quantification of the DNA content isolated from LOs cultured for 10 or 29 days, by spectrophotometry. For each time point, results relative to three independent LOs are reported (means ± SD are also indicated). The symbol ‘ns’ refers to *p* > 0.05 (unpaired Mann–Whitney U test). (**E**,**F**) Histochemical detection of SA-β-gal activity performed on frozen sections of LOs cultured for 10 or 29 days. Quantification of SA-β-gal^+^ cells per time point is reported (**E**). Results expressed as number of positive cells per mm^2^ represent mean ± SD relative to at least three independent LOs per time point. The symbol ‘ns’ refers to *p* > 0.05 (unpaired Mann–Whitney U test). Representative images (left panels: 10×; scale bars = 250 μm) and magnifications of highlighted areas (right panels: 40×; scale bars = 50 μm) are reported (**F**). White arrows point to SA-β-gal^+^ cells. (**G**) Relative gene expression of CDKN1A in LOs cultured for 29 days compared to LOs cultured for 10 days, analyzed by qPCR. For each time point, results relative to three independent LOs are reported (means ± SD are also indicated). The symbol ‘ns’ refers to *p* > 0.05 (unpaired Mann–Whitney U test). (**H**) Cell viability in LOs cultured for 10 or 29 days, by resazurin reduction assay. Results are expressed as percentage values over the mean value relative to day 10 (set as 100%) and represent mean ± SD relative to three independent LOs per time point. The symbol ‘ns’ refers to *p* > 0.05 (unpaired Mann–Whitney U test).

Based on the indication of size stability and metabolic characterization, we selected days 10 and 29 as early and late time intervals to conduct a structural and functional analysis of LOs. Due to extended static culture conditions, cells may experience conditions of stress. Thus, we first monitored apoptosis and cellular senescence as cellular responses to stress signals. Regarding apoptosis, we found no significant alterations in the gene expression of PUMA between days 10 and 29 (Figure 3A). The gene PUMA encodes a BH3-only protein acting as a key mediator of p53-dependent apoptosis [55]. In response to numerous stressors (e.g., nutrient depletion, hypoxia, oxidative stress, DNA damage), p53 activates the transcription of pro-apoptotic genes, such as PUMA or NOXA, with the corresponding proteins leading to cell death [56,57,58]. It has also been reported that PUMA transcription can be activated by p53-independent cell-death pathways, thus showing its central role in apoptosis signaling [59]. Our data suggest that an extended culture did not activate the pro-apoptotic protein PUMA in LOs. After this preliminary indication, we also investigated cleaved-PARP protein levels at both time intervals, finding no statistical differences between days 10 and 29 (Figure 3B,C). PARP cleavage generated by the caspases-3 and -7 is an early hallmark of apoptosis, and when it occurs, cell death is irreversible [60,61,62,63]. Thus, we exclude any possible reduction in the cell population within LOs due to apoptosis activation. In line with this evidence, the DNA content of LOs cultured for 10 or 29 days, a parameter used for a relative estimation of the cell number over time, remained relatively constant with no statistical differences between days 10 and 29, further indicating a preservation of the cell population (Figure 3D). Differently from apoptosis, leading to the loss of damaged cells as a drastic response to tissue degeneration, cellular senescence is an alternative status triggered by persistent stress signals, thanks to which damaged cells and their relative functions are conserved [64]. In fact, senescent cells are non-proliferating but viable cells, characterized by generally irreversible cell-cycle arrest, senescent associated secretory phenotype (SASP), macromolecular damage, and deregulated metabolism (reviewed in Gorgoulis et al. [65]). Since our data excluded apoptosis activation in response to extended culture conditions, we wondered if cellular senescence occurred within LOs. Histochemical detection of SA-β-gal activity revealed a reduction in positive cells in LOs on day 29 compared to day 10, even though it was not significant (Figure 3E,F). The SA-β-gal is a very common biomarker used for assessing cellular senescence in culture and in vivo [66]. However, a multi-marker approach combining more markers has been proposed for an accurate detection of senescent cells [65]. Based on this indication, we also measured the transcript level of CDKN1A (encoding p21^WAF1/Cip1^), observing no significant alterations on day 29 compared to day 10 (Figure 3G), which was in line with the content of SA-β-gal^+^ cells. Under stress, CDKN1A gene expression is up-regulated, and p21^WAF1/Cip1^ induces transiently cell-cycle arrest by inhibiting cyclin-dependent kinases. When the cellular insult is prolonged, p21^WAF1/Cip1^ accumulates, provoking a durable growth arrest and mediating cellular senescence via p53-dependent and -independent pathways [64,67]. Consequently, we can conclude that cellular senescence, along with apoptosis, did not affect the LO cell population over the entire culture period, and homeostatic conditions were established already on day 10 and maintained until day 29. In support of that, no significant differences in the global cell viability of LOs were found between days 10 and 29 (Figure 3H). Nonetheless, a necrotic phenomenon within the LOs cannot be excluded, as Ramachandran et al. evidenced in assembled LOs cultured under static conditions after 72 h [19]. In accordance with this evidence, Mattei et al. computationally predicted the oxygen concentration profile within the same 3D model, finding values below the critical vital concentration in the central part [68]. In our model, by performing H&E and immunofluorescence staining in frozen sections of LOs cultured for 29 days, we observed some limited areas in the internal LO core showing cell loss, presumably indicating necrosis (Supplementary Figure S3A–C).

#### 3.1.4. Histological and Phenotypic Analyses of LOs: Evidence of a Tissue Characterized by Hepatocyte-Derived Primitive Bile Canalicular Networks and LSEC-Formed Tube-like Structures

**Figure 4:**
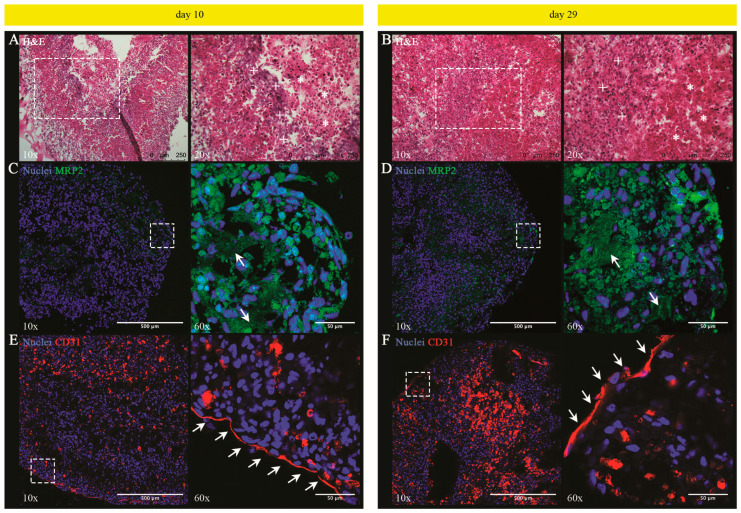
Histological and phenotypic analyses of LOs over time. (**A**,**B**) H&E staining performed on frozen sections of LOs cultured for 10 (**A**) or 29 (**B**) days. Representative images (left panels: 10×; scale bars = 250 μm) and magnifications of highlighted areas (right panels: 20×; scale bars = 250 μm) are reported. Symbols ‘+’ and ‘*’ indicate clusters of elongated and cuboidal cells, respectively. (**C**–**F**) Immunofluorescence staining for the detection of MRP2 (green; **C**,**D**) and CD31 (red; **E**,**F**) in frozen sections of LOs cultured for 10 (**C**,**E**) or 29 (**D**,**F**) days. Representative images (left panels: 10×; scale bars = 500 μm) and magnifications of highlighted areas (right panels: 60×; scale bars = 50 μm) are reported. Nuclei are stained by Hoechst 33,342 (blue). White arrows point to intercellular MRP2^+^ (**C**,**D**) or tube-like CD31^+^ structures (**E**,**F**).

We performed histological and phenotypic analyses on days 10 and 29. Hematoxylin and Eosin (H&E) staining revealed an almost compact tissue, which, on day 10, is already characterized by clusters of cuboidal or elongated cells, and shows no appreciable macroscopic variations in the tissue architecture with respect to day 29 (Figure 4A,B). In parallel, immunofluorescence staining with cell-type specific antibodies revealed MRP2^+^ and CD31^+^ cells, respectively, indicating the presence of UHHs and LSECs that were arranged in distinct clusters after both 10 and 29 days of static culture (Figure 4C–F). In particular, MRP2 (also known as ABCC2) is an efflux pump located in the apical (canalicular) membrane of polarized hepatocytes, where it is involved in the excretion of numerous conjugates of endo- and xenobiotics substances into the bile [69,70,71]. It has been reported that MRP2/ABCC2 is relatively well preserved in UHHs compared to PHHs [72]. Interestingly, our results also show that UHHs merged their apical domains to form continuous MRP^+^ structures resembling primitive bile canalicular networks already after 10 days (Figure 4C, right panel). At longer time points (i.e., day 29), these structures appeared more widespread (Figure 4D, right panel). On the other side, CD31 (also termed PECAM-1) is a well-known marker for vascular endothelial cells, where it participates in cell–cell adhesion [73]; however, its expression and relevance are controversial in the case of LSECs. In fact, numerous studies showed CD31 is poorly/less expressed in the normal liver, with an increase in fibrotic livers [73,74,75]. The increase in CD31 expression is commonly associated with LSECs de-differentiation (a phenomenon called capillarization), which determines the loss of the typical fenestrae and the formation of an organized basement membrane, occurring in vivo at the onset of liver fibrosis or in vitro cultures of LSECs [76,77,78]. In contrast, Neubauer et al. reported that CD31 expression had no variations between normal and damaged livers in rats and humans [79]. Interestingly, it has been demonstrated in rats that CD31 is localized in the cytoplasm of normal LSECs, whereas it is present on the cell membrane when LSECs are capillarized [78]. Similarly, it has been reported that LSECs in the livers of HCV-infected patients maintain their specific phenotype and exhibit a cytoplasmic localization of CD31 [80]. Hence, it is reasonable that the different signal of CD31 obtained from normal and fibrotic livers could be due to the different localization of this marker in LSECs and, thus, to inadequate staining procedures (e.g., including or not cell permeabilization), even though a definitive demonstration has not yet been provided. Immunofluorescence analysis reported herein was performed on permeabilized cells and shows the expression of CD31 by LSECs at both selected time intervals (Figure 4E,F; see magnifications on the right panels). The reported data, however, cannot clearly discriminate cytoplasmic or superficial localization of this marker, so that we cannot exclude the superficial localization of CD31 and, thus, the capillarization of these cells. In this framework, Kaden et al. recently detected both intracellular and membrane-associated CD31 fractions in three different donors of upcyte^®^ LSECs, including donor #462 used herein [81]. Similar to MRP2^+^ UHHs forming intercellular structures in LOs, CD31^+^ LSECs arranged to form tube-like structures, mainly localized at the periphery, at both time intervals (Figure 4E,F). Such tube-like structures were also positively stained for VE-cadherin (Supplementary Figure S4A–F and Video S1), which is a transmembrane protein forming adherens junctions (AJ) between vascular endothelial cells and, thus, contributing to maintaining the vascular endothelium integrity in vivo [82,83,84,85]. In LSECs, the effective expression of VE-cadherin has been debated. However, Ding et al. operationally described murine LSECs as a phenotypically and functionally defined niche of VEGFR3^+^CD34^−^VEGFR2^+^VE-cadherin^+^FactorVIII^+^CD45^−^ endothelial cells [86]. Similarly, Géraud et al. proved that rat and human LSECs exhibit specific intercellular junction complexes containing VE-cadherin [87]. In accordance with the literature, our results indicate the expression of VE-cadherin mainly by cells forming tube-like structures in LOs. Ramachandran et al. reported a comparable spatial distribution of hepatocytes and endothelial cells after 72 h and 10 days of culture under dynamic conditions within an analogous 3D liver model [19]. Our results are in line with these data and even progress beyond the state of the art as we investigate a longer culture period (29 days), providing additional structural insights on macrostructures resembling bile canaliculi and liver sinusoids within LOs.

#### 3.1.5. UHHs in LO Retain the Hepatocellular Functionality until Day 29

**Figure 5:**
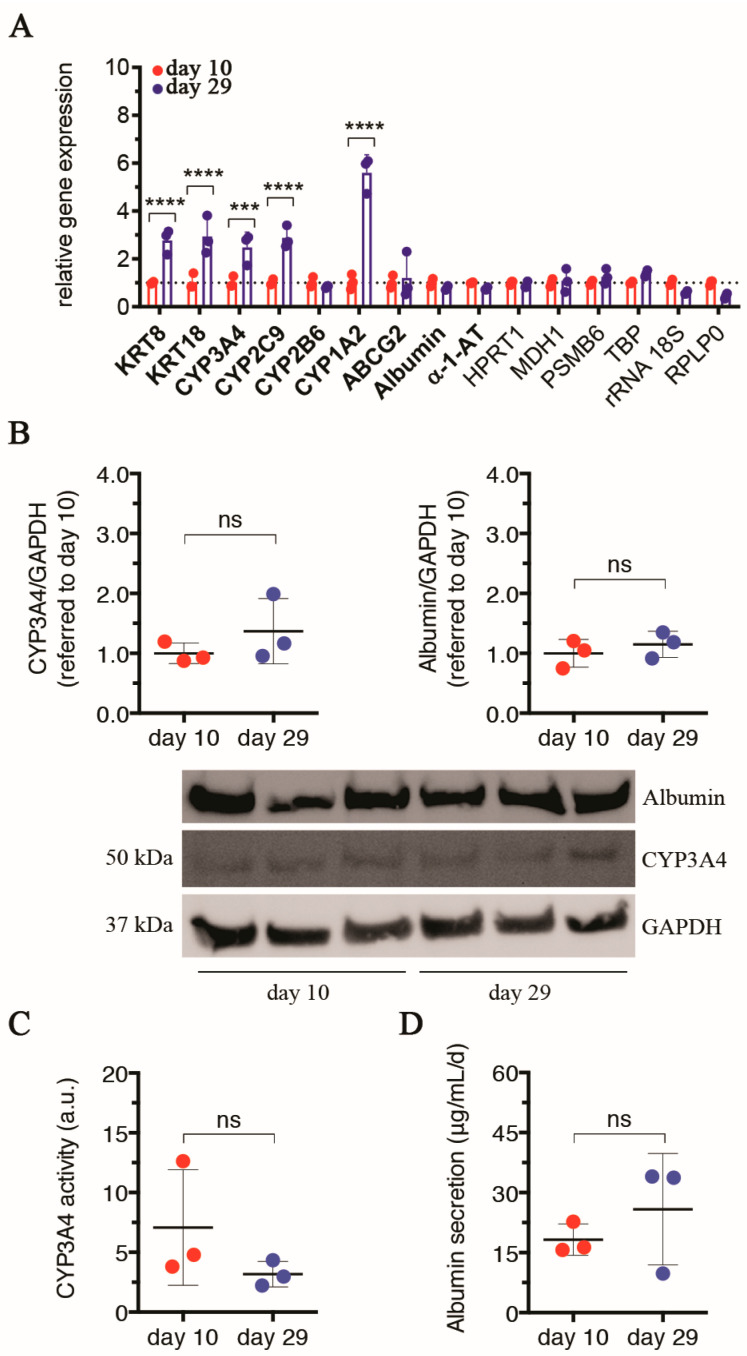
Functional stability of LOs over time. (**A**) Relative gene expression of some hepatocyte-specific markers and six endogenous control genes in LOs cultured for 29 days compared to LOs cultured for 10 days, analyzed by qPCR. Results are expressed as mean ± SD of three independent LOs per time point. The symbols ‘***’ and ‘****’ refer to *p* = 0.0001 and *p* < 0.0001, respectively (two-way ANOVA). (**B**) CYP3A4 and Albumin protein levels in LOs cultured for 29 days compared to day 10, analyzed by Western blot. For each marker, the blot relative to three independent LOs per time point probed with specific primary antibodies (lower panels) and densitometric analyses of band intensities (upper panels) are reported. GAPDH was used as internal control. Results are expressed as n-fold increase over the mean value relative to day 10. The symbol ‘ns’ refers to *p* > 0.05 (unpaired Mann–Whitney U test). (**C**) BFC-metabolizing activity of CYP3A4 in LOs cultured for 29 days compared to day 10. Results are expressed as mean ± SD of three independent LOs per time point. The symbol ‘ns’ refers to *p* > 0.05 (unpaired Mann–Whitney U test). (**D**) Albumin secretion by LOs cultured for 29 days compared to day 10. Results are expressed as mean ± SD of three independent LOs per time point. The symbol ‘ns’ refers to *p* > 0.05 (unpaired Mann–Whitney U test).

A relatively wide amount of literature is currently available on the hepatocellular functionality of UHHs when cultured in conventional 2D monolayers. In particular, differentiated UHHs showed stable activity of representative phase-I CYPs and prolonged activity of the phase-II UGT for up to 14 or 21 days [88], as well as stable production of Albumin that was monitored for 10 days [4]. Moreover, it has been reported that a sandwich configuration significantly increased the basal gene expression and activity of some CYPs compared to monolayer UHH cultures for at least 14 days [89]. Analogously, sandwich cultures determined an up-regulated expression of sinusoidal solute carrier transporters [72]. Overall, based on these data, it is plausible that UHHs are functionally stable for approximately 2 or 3 weeks in either monolayer- or sandwich-based cultures. Herein, we aimed to investigate the functional stability of UHHs within our tri-lineage 3D model, where the initial representativeness of these cells is approx. 45.5% of UHHs, approx. 45.5% of LSECs, and approx. 9% of hbmMSCs. First, we analyzed over time a range of genes involved in the phenotypic specification of UHHs (KRT8/18) and some specific hepatocellular functions (CYP3A4, -2C9, -2B6, -1A2, ABCG2, Albumin, and α-1-AT) by qPCR. On day 29 compared to day 10, we found an up-regulated gene expression of KRT8/18 (*p* < 0.0001 for both the genes), as well as of three out of four CYPs tested (i.e., CYP3A4, -2C9, and -1A2; *p* ≤ 0.0001; Figure 5A). In parallel, no appreciable transcription alterations were found for CYP2B6, the efflux transporter ABCG2, and the plasma proteins Albumin and α-1-AT. The differences quantified for gene expression between days 10 and 29 were statistically evaluated including endogenous controls (i.e., HPRT1, MDH1, PSMB6, TBP, rRNA 18S, and RPLP0) to substantiate the gene activation reported above. Taken together, our data indicate a relatively stable transcriptional profile of UHHs over the extended culture of LOs.

Afterward, we selected as representative markers of functional hepatocytes CYP3A4 and Albumin, and for them, we also measured the intracellular protein levels at both time intervals. For each marker, no statistical variation was found on day 29 compared to day 10 (Figure 5B). As a further confirmation, we assessed the metabolic activity of CYP3A4, observing a constant activity between days 10 and 29 (Figure 5C). These results indicate that despite differences in CYP3A4 transcription (Figure 5A), post-transcriptional regulations might compensate for the differences in gene expression to ensure a preserved CYP3A4 functional activity. Furthermore, the secreted amount of Albumin was comparable between the two days of observation (Figure 5D).

Hence, from the presented data, we conclude that UHHs show preserved relatively stable activity of two key hepatic markers (CYP3A4 and Albumin) for the entire LO culture (until 29 days) at the applied experimental conditions. In normal livers of adult Caucasians, CYP3A4 is the most abundant enzyme of the CYP family and is responsible for the phase-I metabolism of approximately 50% of therapeutic drugs [90,91]. Albumin is primarily synthesized by hepatocytes and, being the most abundant serum protein, regulates numerous functions, such as maintaining the colloidal osmotic pressure of blood or transporting ions and endogenous molecules/drugs in the bloodstream [92]. Our results also indicate an advancement with respect to the state of art since Ramachandran et al. previously reported the expression of some functional genes only within 10 days of incubation of a liver organoid comparable to LOs used herein [19]. Interestingly, the authors reported the expression of CYP2A6, -2D6, -2E1, UGT1A3, SULT1A1, and Albumin comparable to the levels found in PHHs or whole livers derived from different donors. Similarly, the basal activity of CYP3A4, -2B6, and 2C9 was comparable to or even higher than the ones exhibited by PHHs or in UHHs cultured in LOs for 10 days [19].

Overall, the body of data reported in this work (i.e., dimensional stability starting from day 7, see Figure 1; stable glucose and amino acid consumption in the temporal window between days 15 and 29, see Figure 2; no activation of apoptosis or cellular senescence on day 29 compared to day 10, see Figure 3A–C,E–G; no variation in global cell viability on day 29 compared to day 10, see Figure 3H; no alterations or even an improvement of hepatocellular functions on day 29 vs. day 10, see Figure 5) clearly supports that the developed LOs exhibited homeostatic conditions in the temporal window between days 10 and 29 and, thus, can be used as stable cellular systems (internal control) to compare against treated LOs in the downstream investigations (for example, with toxicants). Specifically, the good performance of UHHs within LOs for longer culturing time intervals (more than 4 weeks of culture), hence, paves the way to investigations on hepatocellular functionality alterations, which can be based on prolonged exposures of LOs to toxicants as, for example, nanomaterials.

### 3.2. Repeated Exposure of LOs to GO and Toxicological Impact

#### 3.2.1. Physical-Chemical Characterization of GO in LOM Medium and Evaluation of Penetration by Histological Analysis

**Figure 6:**
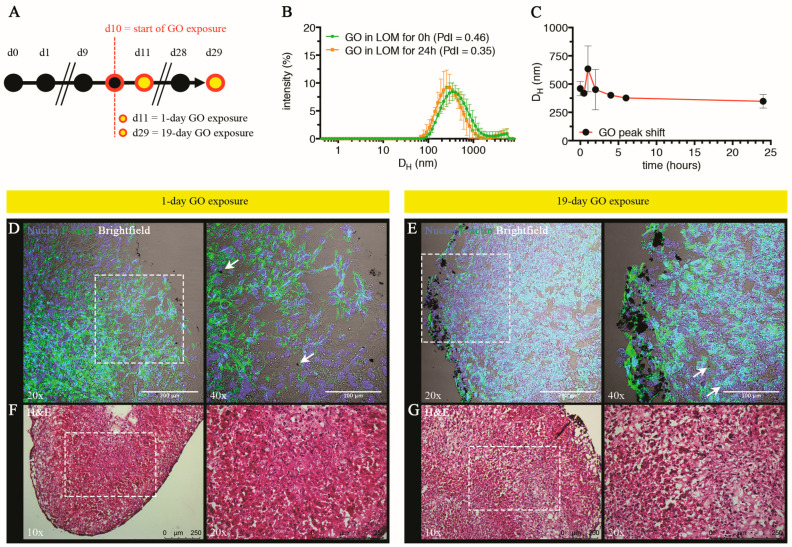
Single and repeated LO exposure to GO. (**A**) Experimental schematic used for the treatment of LOs with GO. After LO maturation (first 10 days of culture), LOs were treated for 1 or 19 days using single or repeated daily doses (i.e., 40 μg/mL GO every 24 h). (**B**) Size distribution profiles of 40 μg/mL GO in LOM for 0 (green curve) and 24 h (orange curve) at 37 °C, by DLS analysis. (**C**) Dimensional shift of GO peak position over time by consecutive DLS measurements (up to 24 h), showing changes in GO mean size. “DH” means the hydrodynamic diameter of particles measured by DLS analysis. (**D**,**E**) Visualization of GO aggregate/deposit penetration carried out on frozen sections of LOs treated with 40 μg/mL GO for 1 (**D**) or 29 (**E**) days, by conventional brightfield optics. Representative images (left panels: 20×; scale bars = 200 μm) and magnifications of highlighted areas (right panels: 40×; scale bars = 100 μm) are reported. Nuclei and F-actin cytoskeleton are stained by Hoechst 33,342 (blue) and phalloidin (green), respectively. White arrows point to GO aggregates (black). (**F**,**G**) H&E staining performed on frozen sections of LOs treated with 40 μg/mL GO for 1 (**F**) or 29 (**G**) days. Representative images (left panels: 10×; scale bars = 250 μm) and magnifications of highlighted areas (right panels: 20×; scale bars = 250 μm) are reported.

Herein, we exposed LOs to single or repeated GO doses for 19 days and followed the relative impact on cell viability and hepatocellular functionality. We selected single doses to simulate acute exposures, whereas we applied a repeated dosing regimen to better appreciate a likely progressive accumulation of GO in the liver as it may potentially occur in real-life exposures (for instance, occupational settings [31,93,94,95,96,97]). Figure 6A reports the applied experimental schematic according to which, upon achievement of organoid maturation at day 10, LOs were exposed to single or repeated daily doses of 40 μg/mL GO till day 29 (see Section 2 for further technical details).

Freshly prepared GO suspensions were added every 24 h according to the selected dosing regimen (Figure 6A); we assessed their colloidal stability by selecting the same temporal window (namely 24 h) during which GO suspension experiences interactions with the LOs within the medium (i.e., LOM). DLS analysis revealed that, as opposed to the control condition (namely GO-free LOM suspensions), 40 μg/mL GO was present in the suspension as agglomerates of approximately 500 nm (Figure 6B and Figure S5A), which remained stably suspended for 24 h (Figure 6C). However, apart from soluble agglomerates, we also observed visible aggregates, which sedimented on the bottom (Supplementary Figure S5C); this indicates that the effective dose stably suspended in LOM is lower than 40 μg/mL, although (by means of the techniques employed) we cannot extrapolate the exact value. The tendency of GO to agglomerate/aggregate in other culture media (e.g., in complete HHPM) has already been reported by our group [28]. Furthermore, we analyzed the changes in the surface charge of GO upon 24 h incubation in LOM by zeta potential analysis. We found an increase in the surface charge compared to GO dispersed in Milli-Q^®^ water from approx. −44 mV to −28 mV that was almost stable over time (Supplementary Figure S5B). This effect can be possibly ascribed to the formation of a bio-macromolecular corona around GO [98] and is in line with previous observations in other cellular media [28].

We observed frozen sections of GO-treated LOs stained for F-actin cytoskeleton (green) and counterstained for nuclei (blue) by brightfield optics: we found in the outer LO shell the presence of large GO deposits, which appeared more numerous after 19 days of repeated exposure than those observed after the single dose. Moreover, we also detected very few micrometer-sized aggregates within the tissue, presumably indicating a certain penetration of GO into the LOs (Figure 6D,E). Based on the presented data, we cannot exclude the diffusion of smaller GO aggregates, which are impossible to visualize due to the low resolution of the technique used. H&E staining of treated LOs revealed no macroscopic insults to the tissue after single or repeated exposure to GO (Figure 6F,G). In conclusion, our results indicated that GO is taken up by LOs, although by the employed technique we cannot derive an exact quantification. We can speculate that, due to the spatial organization of cells in LOs, the uptake is lower than in 2D-cultured cells. Scientific evidence in support of this observation is provided, for example, by the scarce penetration of GO (25 μg/mL) in liver spheroids of murine Hepa1–6 and KUP5 cells treated for 16 h, that showed a predominant localization in the outer cellular layer [99]. Moreover, a similar trend was also observed for other nanomaterials (e.g., V_2_O_5_ and TiO_2_) and other 3D tumor models.

Overall, these findings indicate that although the uptake of NMs in 3D cell models is influenced by the specific physical-chemical properties of the NM, the intrinsic morphology of the tissue mimicked by the 3D model may better resemble what happens in the organ, acting as a passive and active physical barrier [100,101,102,103,104]. In this regard, in vivo data in a mouse model clearly evidenced that GO accumulated in the liver 24 h after a single administration [105]. However, the authors observed a differential interlobular localization of GO with a preferential presence around the portal triad compared to the central vein in mice exposed for 7 days. Considering that the bloodstream flows from the portal triad to the central vein through fenestrated sinusoids, such a zonal accumulation of GO may indicate that penetration of GO within the hepatic lobules is impeded.

#### 3.2.2. Graphene Oxide Does Not Induce Cytotoxicity in Liver Organoids upon Single or Repeated Exposure

**Figure 7:**
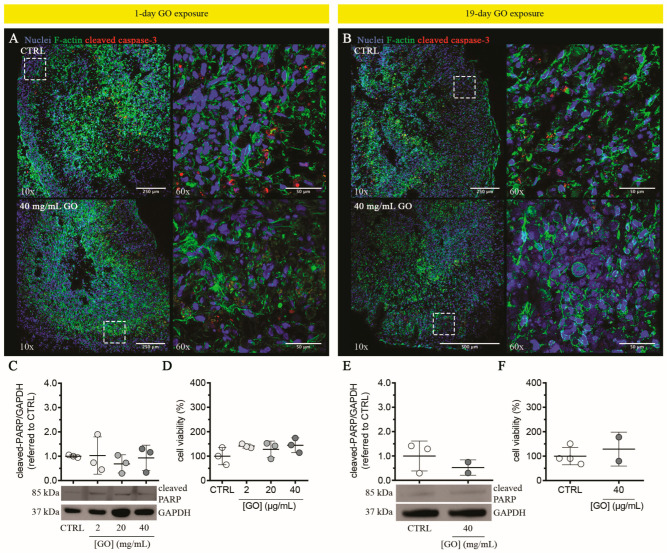
Cytotoxicity in LOs upon single and repeated GO exposure. (**A**,**B**) Immunofluorescence staining for the detection of cleaved caspase-3 (red) in frozen sections of LOs treated or not with 40 μg/mL GO for 1 (**A**) and 19 (**B**) days. “CTRL” refers to un-treated control LOs. Representative images (left panels: 10×; scale bars = 250 μm) and magnifications of highlighted areas (right panels: 60×; scale bars = 50 μm) are reported. Nuclei and F-actin cytoskeleton are stained by Hoechst 33,342 (blue) and Phalloidin (green), respectively. (**C**,**E**) Cleaved-PARP levels in LOs treated or not with 2–40 μg/mL GO for 1 (**C**) and 19 (**E**) days, analyzed by Western blot. Densitometry analysis of band intensities relative to two or more independent LOs per experimental condition (upper panels) and representative blots (lower panels) are reported. GAPDH was used as internal control. Results are expressed as n-fold increase over the mean value relative to CTRL and represent means ± SD. (**D**,**F**) Cell viability in LOs treated or not with 2–40 μg/mL GO for 1 (**D**) and 19 (**F**) days, by resazurin reduction assay. Results are expressed as percentage values over the mean value relative to CTRL (set as 100%) and represent mean ± SD relative to two or more independent LOs per experimental condition.

We assessed cytotoxicity induced by single or repeated exposures of GO (2–40 μg/mL). Following both exposures, 2–40 μg/mL GO induced no evident activation of cleaved caspase-3 in treated LOs compared to un-treated control (Figure 7A,B; left panels). In particular, magnifications of the outer shell of treated LOs, corresponding to the zones where GO was visible (Figure 6D,E), showed no qualitative differences in the cleaved caspase-3 signal compared to the controls (Figure 7A,B; right panels). Similarly, protein levels of cleaved PARP did not vary among samples (GO-treated LOs vs. un-treated controls) (Figure 7C,E). Overall, these data indicate that GO exposure does not induce apoptosis in LOs, independently of the exposure extension. In support of such a lack of toxicity, resazurin reduction assay revealed negligible effects of GO on cell viability of LOs treated under the same experimental conditions (i.e., 2–40 μg/mL GO single or repeated exposure; Figure 7D,F). In contrast, acute GO exposure showed cytotoxic activity against 2D monolayer cultures of different hepatic cells (HepG2 cells and UHHs), with IC_50_ values in the range of 50–100 μg/mL [28,106,107]. Also, mild apoptosis activation with no induction of PARP cleavage in UHHs [28] upon acute stimulation with 20–80 μg/mL GO, and an increase in early apoptotic cells in HepG2 cells [107] after 24 h of treatment with up to 50 μg/mL GO have been observed.

#### 3.2.3. Graphene Oxide Down-Regulates CYP3A4 in Liver Organoids upon Repeated Exposure

**Figure 8:**
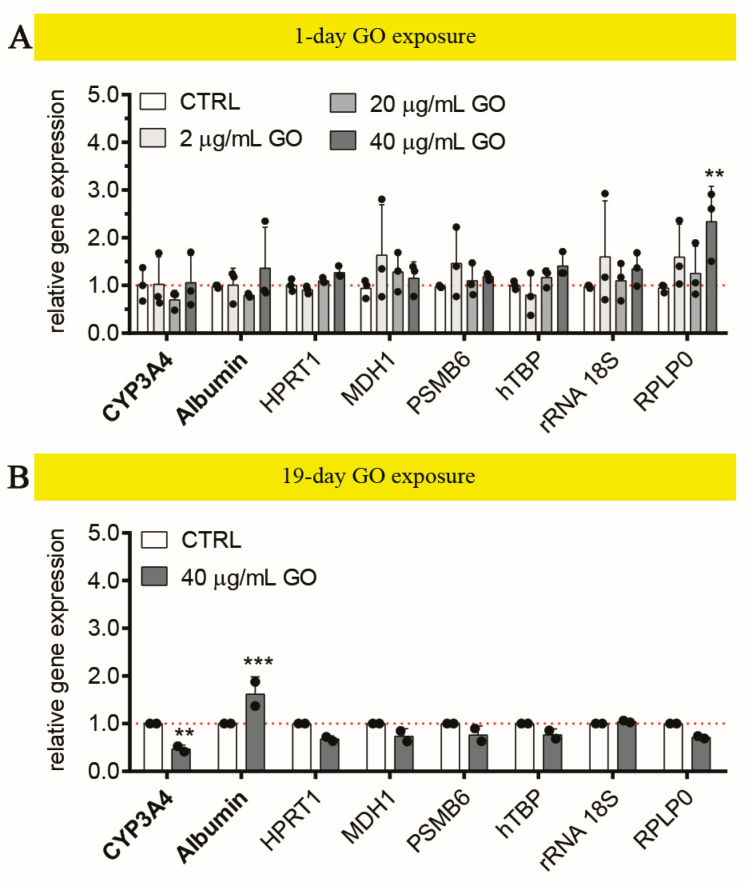
Hepatocyte-specific response of LOs upon single and repeated GO exposure. (**A**,**B**) Relative gene expression of CYP3A4 and Albumin, along with six endogenous control genes, in LOs cultured for 10 days and treated with 2–40 μg/mL GO for 1 (**A**) and 19 (**B**) days, analyzed by qPCR. Results are expressed as mean ± SD of two or more independent LOs per experimental condition. The symbols ‘**’ and ‘***’ refer to *p* ≤ 0.0026 and *p* = 0.0005, respectively (two-way ANOVA).

We investigated the hepatocellular response of LOs exposed to single and repeated GO exposures, focusing on CYP3A4 and Albumin, which were hepatocellular markers deeply characterized in un-treated LOs (see Figure 5) and, previously, in models of 2D UHH cultures [28]. Upon a single treatment (up to 40 μg/mL GO), no statistically significant variations in the gene expression of both markers were induced by GO, also according to the modulation of a wide range of quantified endogenous control genes (i.e., HPRT1, MDH1, PSMB6, TBP, rRNA 18S, and RPLP0; Figure 8A). Interestingly, we observed a mild but significant up-regulation of RPLP0 (*p* = 0.0013), which contributes to forming the ribosome 60S subunit and is involved in protein translation. Few literature data indicate that the up-regulation of RPLP0 is related to stress conditions [108,109], thus suggesting that GO can induce general cellular stress, which, however, did not lead to specific functional alterations of LOs after 24 h of exposure. On the other hand, the repeated 19-day treatment with 40 μg/mL GO significantly determined a 0.5-fold down-regulation of CYP3A4 with respect to un-treated LOs (*p* = 0.0026) and a 1.6-fold up-regulation of Albumin (*p* = 0.0005; Figure 8B), even though an unaltered intracellular protein amount of Albumin with respect to the control was observed (Supplementary Figure S6B). The GO impact on the CYP system and Albumin has been reported in the literature using different approaches based on 2D in vitro systems (HepaRG^TM,^ microsomal models expressing CYP isozymes, UHHs); however, the doses applied were lower and in single acute exposure (range 3–100 μg/mL). In particular, Strojny et al. reported a reduced transcription of CYP3A4, -2B6, -1A2, and -2E1 in differentiated HepaRG^TM^ cells after 24 h of treatment with 50 μg/mL GO [110]. We previously showed in UHHs that sub-lethal GO concentrations (up to 80 μg/mL) inhibited both the gene expression and metabolic activity of CYP3A4, CYP2C9 along with the down-regulation of CYP2B6 and -1A2. In addition, we found a reduced intracellular level of Albumin upon 24 h exposure [28]. Therefore, by comparing these data (single vs. repeated doses in 2D vs. 3D cell systems; Figure 6E vs. Figure 7B,E,F), we can conclude that LOs appear less sensitive to GO exposure with respect to 2D cultures. Indeed, we did observe alteration of CYPs enzymes even though only upon 19-day repeated exposure. This evidence further confirms that the intracellular accumulated dose is lower than that applied to 2D cell models. A similar trend is also observed with the soluble drug Rifampicin, a well-known inducer of CYP3A4. In this case, LOs, upon maturation, were treated with 100 μM Rifampicin/day for 3 consecutive days. Here, we found an approximately 5-fold up-regulation of CYP3A4 (*p* < 0.0001; Supplementary Figure S6A). Conversely, when monolayer-grown UHHs were treated with 50 μM Rifampicin/day for 3 consecutive days, a stronger up-regulation of CYP3A4 was observed (41-fold compared to the control gene expression) [28]. These data further confirm that LOs uptake is reduced compared to 2D cell systems. In any case, the observed changes can be interpreted as a clear and real toxic effect on liver. Notably, studies in mice exposed for 7 days to GO have also confirmed GO-induced CYP alterations. Wu et al. found reduced expression of CYP7A1, -1A1, and -3A7, especially around the portal triad, where GO preferentially accumulated [105]. These data highlight the reliability of the developed tri-lineage 3D LOs as an in vitro tool for toxicity assessment of nanomaterials.

## 4. Conclusions

Here, we report the production of human multi-lineage liver organoid model (LO) and its structural and functional assessment over a period of 29 days. LOs were characterized in terms of size stability, energy metabolism, cytotoxicity (e.g., apoptosis and senescence), spatial organization of cells, and expression of hepatocellular markers. Our results indicate that LOs retain good viability, mainly supported by no signs of apoptosis or cellular senescence activation, and exhibit an architecture characterized by primitive hepatic structures (i.e., bile canaliculi and liver sinusoids), resembling their counterparts in vivo for extended times (up to 29 days). Interestingly, they also conserve a sustained gene expression of representative CYPs and transporters, and a stable production of Albumin until the end of the culture. Based on these structural and functional features, LOs can be considered good in vitro models for pre-clinical assessment of toxicants, for which the incorporation of multiple differentiated cell types, their organization in a 3D structure, and the maintenance of hepatocellular functionality for at least 2 weeks are fundamental requirements [10]. Moreover, according to OECD indications, an exposure regimen reflecting acute or repeated doses for 28 days is requested for testing toxicants (OECD Test No. 407 [20]). Our LOs presented features, such as culture stability over approximately 1 month and the feasibility of repeated daily exposure, which potentially allows testing of toxic substances (such as nanomaterials). Accordingly, we assessed the GO impact on LO hepatic functionality. We exposed mature LOs to single or repeated GO doses (40 μg/mL) for up to 19 days, finding no macroscopic tissue damage or apoptosis activation upon both exposure protocols. However, as opposed to single exposure, repeated exposures did alter CYP3A4 gene expression, which appeared down-regulated. Notably, CYP3A4 down-regulation was found in mice exposed to GO [105], supporting the idea that LOs can be suitably considered alternative or complementary in vitro systems to the animal models. Indeed, when comparing the LO response to 2D cell models, repeated exposures are required to induce CYP alterations in the 3D model. We speculate that a complex cell arrangement acts as a compact physical barrier hindering GO penetration, as already observed in 3D tumor models upon nanomaterial exposure [99].

Overall, our study provides valuable insights into GO hepatotoxicity using an advanced liver model and an exposure regimen mimicking real-life exposures and sub-chronic liver accumulation. As a future perspective, our approach, described herein, will be advanced with the inclusion in LOs of the body’s immune system (e.g., Kupffer cells), the detection of relevant liver biomarkers (e.g., ALT, AST), and the use of dynamic perfused systems (e.g., organ-on-a-chip models) to construct even more accurate pre-clinical liver models for hepatoxicity prediction of chemical compounds and nanomaterials.

## Data Availability

The original contributions presented in the study are included in the article/Supplementary Materials, further inquiries can be directed to the corresponding author.

## Supplementary Materials

The following supporting information can be downloaded at: https://www.mdpi.com/article/10.3390/cells13181542/s1, Table S1: Primer pairs used for the gene expression analysis by qPCR. Figure S1: (A) Representative bright-field images by optical microscopy of cell morphology of UHHs, LSECs, and hbmMSCs mixed at the ratio of 1:1:0.2, respectively, to generate LOs (scale bar = 500 μm). (B) Representative morphology of LOs. Each LO is cultured with LOM in Matrigel®-coated 24-well plates, under static conditions. “LOM” refers to the liver-organoid medium. (C) Photographs of LOs cultured at different time intervals ranging from 4 h to day 29. (D) Schematic used for measuring the 2D projection area of LOs at defined time intervals by Adobe Photoshop and Fiji (for technical details, see Section 2). Figure S2: (A) Lactate to glucose molar ratio relative to LOs at defined time intervals (day 1, 2, 3, 7, 10, 15, 20, 25, and 29). For each time point, results represent means ± SD of eight independent LOs. (B) Lactate to glucose molar ratio relative to UHHs cultured in a 2D format for 10 days under conditions similar to those used for LOs (i.e., 1.0 × 106 cells in LOM). For each time point, results represent means ± SD of four replicates. The symbols ‘*’ and ‘ns’ refer to *p* = 0.0142 and *p* > 0.05, respectively (ordinary one-way ANOVA). Figure S3: (A) H&E staining performed on frozen sections of a representative LO cultured for 29 days (5×; scale bar = 750 µm). The highlighted area indicates presumably necrotic tissue. (B,C) Immunofluorescence staining for the detection of nuclei (stained blue by Hoechst 33342) in different frozen sections of the same LO stained by H&E. Large images created combining more 10× acquisitions automatically are reported (scale bars = 500 µm). The highlighted areas indicate presumably necrotic tissue across sections. Figure S4: (A) Immunofluorescence staining for the detection of VE-cadherin (red) in frozen sections of LOs cultured for 10 (left panel) or 29 days (right panel). Representative images (60×; scale bars = 50 μm) are reported. Nuclei are stained by Hoechst 33342 (blue). White arrows point to intercellular tube-like structures. (B) Representative high-magnification image (60×; scale bars = 50 μm) after stack reconstruction of a portion of the organoid, acquired by confocal microscopy and stained for VE-cadherin (red) and nuclei (Hoechst 33342, blue). White dotted lines indicate the tube-like structures. (C) Representative high-magnification image (60×; scale bars = 50 μm) of a single plane from the image stack, showing VE-cadherin-positive linear structures. (D–F) Single-channel images with inverted colors relative to the image shown in panel C, displaying the presence of nuclei and VE-cadherin markers, respectively. The dotted green lines in panel F trace the tube-like structures highlighted in panel B. Video S1: The animation presents a representative high-magnification image (60×) after stack reconstruction of a portion of the organoid, acquired using confocal microscopy. The sample is stained for VE-cadherin (red) and nuclei (Hoechst 33342, blue). The video sequence is as follows: it begins with a composite colour image showing the three-dimensional distribution of both nuclei and VE-cadherin signals. The second segment features a single-channel reconstruction with inverted colours, highlighting the cell nuclei. The final segment displays a single-channel reconstruction with inverted colours, illustrating the VE-cadherin distribution and the presence of tubular structures. Figure S5: (A) Size distribution profiles of GO-free LOM, by DLS analysis. (B) Surface charge of particle-corona com-plexes after incubation of GO in LOM for 0, 2, and 24 h by Zeta Potential (ZP) analysis. (C) Representative images of LOs treated with 40 μg/mL GO for 1 and 19 days or un-treated (indicated as CTRL). The white arrow points to the treated LO almost completely covered by GO deposits after 19 days of exposure. Figure S6: (A) Relative gene expression of CYP3A4, along with six endogenous control genes, in LOs cultured for 7–10 days and then daily treated with 100 μM rifampicin for up to 3 days or un-treated (indicated as CTRL), analysed by qPCR. Results are expressed as mean ± SD of three independent LOs per experimental condition. The symbol ‘****’ refer to *p* < 0.0001 (two-way ANOVA). (B) Albumin levels in LOs daily treated with 40 μg/mL GO for up to 19 days or un-treated, analysed by western blot. Densitometric analysis of band intensities relative to two or more independent LOs per experimental condition (upper panels) and a representative blot (lower panels) are reported. GAPDH was used as internal control. Results are expressed as n-fold increase over the mean value relative to CTRL and represent means ± SD. The symbol ‘ns’ refers to *p* > 0.05 (unpaired Mann-Whitney U test). Raw Data. Western blot: Original western blots relative to the total protein content from LOs assessed on days 10 and 29 (A) and LOs treated with 2–40 μg/mL GO or untreated (indicated as CTRL) for 1 (B) and 19 days (C). Samples from three independent LOs per experimental condition (except of LOs treated with 40 μg/mL GO for 19 days) were run simultaneously on the same gel, and the corresponding blot was probed consecutively with antibodies raised against CYP3A4, Albumin, cleaved-PARP or GAPDH. Red selections indicate the boundaries whereby the lanes were cropped to be reported in the main text (see Figure 3, panels C; Figure 5 panel B; Figure 7 panels C and E; Figure S6 panel B).

## Abbreviations

2D	two-dimensional
3D	three-dimensional
α-1-AT	α-1-antitrypsin
ABCG2	ATP binding cassette subfamily G member 2
ANOVA	analysis of variance
ATP	adenosine 5′-triphosphate
BCAA	branched-chain amino acid
BFC	7-benzyloxy-4-trifluoromethylcoumarin
BSA	bovine serum albumin
CD31/PECAM-1	cluster of differentiation 31/platelet endothelial cell adhesion molecule-1
CDKN1A	cyclin dependent kinase inhibitor 1A
cDNA	complementary DNA
CoA	coenzyme A
CTRL	un-treated, control condition
CYP	cytochrome P450 (family…subfamily…member…)
D_2_O	deuterium oxide
DH	hydrodynamic diameter
DLS	dynamic light scattering
DMEM	Dulbecco’s modified Eagle medium
DNA	deoxyribonucleic acid
DPBS	Dulbecco’s phosphate buffered saline
ELISA	enzyme-linked immunosorbent assay
F-actin	filamentous actin
GAPDH	glyceraldehyde-3-phosphate dehydrogenase
GO	graphene oxide
H&E	hematoxylin and eosin
hbmMSC	human bone marrow-derived mesenchymal stromal cell
HFC	7-hydroxy-4-trifluoromethylcoumarin
HHPM	hepatocyte high-performance medium
HPRT1	hypoxanthine phosphoribosyltransferase 1
KRT8/18	keratin 8/18
LDH	lactate dehydrogenase
LO	liver organoid
LOM	liver-organoid medium
LSEC	upcyte^®^ liver sinusoidal endothelial cell
LSECm	LSEC culture medium
MDH1	malate dehydrogenase 1
MRP2/ABCC2	multidrug resistance-associated protein 2/ATP binding cassette subfamily C member 2
NASH	non-alcoholic steatohepatitis
NMR	nuclear magnetic resonance
ns	not significant
OECD	organisation for economic co-operation and development
OSM	oncostatin M
OXPHOS	oxidative phosphorylation
PARP	poly(ADP-ribose) polymerase
PC1 or 2	principal component 1 or 2
PCA	principal component analysis
PHH	primary human hepatocyte
PSMB6	proteasome 20S subunit beta 6
PUMA	p53 up-regulated modulator of apoptosis
qPCR	quantitative real time polymerase chain reaction
RNA	ribonucleic acid
ROI	region of interest
RPLP0	ribosomal protein lateral stalk subunit P0
rRNA 18S	ribosomal RNA 18S
SA-β-gal	senescence-associated β-galactosidase
SASP	senescent associated secretory phenotype
SD	standard deviation
TBP	TATA binding protein
TCA	tricarboxylic acid
TSP	trimethylsilylpropanoic acid
UHH	upcyte^®^ human hepatocyte
VE-cadherin	vascular endothelial cadherin
XME	xenobiotic-metabolizing enzyme
