# Supplementary material for: Human Multi-Lineage Liver Organoid Model Reveals Impairment of CYP3A4 Expression upon Repeated Exposure to Graphene Oxide
Source: Cells. 2024 Sep 13;13(18):1542. doi: 10.3390/cells13181542 (PMC11429598; doi:10.3390/cells13181542)
Supplement: Supplementary file 1 [file cells-13-01542-s001.zip › cells-3120377-supplementary.pdf]

Human Multi-Lineage Liver Organoid Model Reveals Impairment of CYP3A4 Expression upon Repeated Exposure to Graphene Oxide

Alessio Romaldini [1,†], Raffaele Spanò [1,†], Marina Veronesi [2,3], Benedetto Grimaldi [4], Tiziano Bandiera [1] and Stefania Sabella [1,*]

[1] Nanoregulatory Group, D3 PharmaChemistry, Istituto Italiano di Tecnologia, via Morego 30, 16163 Genoa, Italy; alessio.romaldini@gmail.com (A.R.); raffaele.spano@iit.it (R.S.)
[2] Structural Biophysics Facility, Istituto Italiano di Tecnologia, via Morego 30, 16163 Genoa, Italy; marina.veronesi@iit.it
[3] D3 PharmaChemistry, Istituto Italiano di Tecnologia, via Morego 30, 16163 Genoa, Italy
[4] Molecular Medicine, Istituto Italiano di Tecnologia, via Morego 30, 16163 Genoa, Italy
* Correspondence: stefania.sabella@iit.it
† These authors contributed equally to this work.

SUPPLEMENTARY TABLE

Table S1 Primer pairs used for the gene expression analysis by qPCR.

Target Gene	Direction	Primer Sequence (5'->3')	Amplicon Size	$T_{annealing}$	E_{target}
PUMA	*forward*	ACGACCTCAACGCACAGTACGA	147 bp	62°C	1.79
	reverse	CCTAATTGGGCTCCATCTCGGG			
CDKN1A	*forward*	AGTCAGTTCCTTGTGGAGCC	108 bp	58°C	1.60
	reverse	CATGGGTTCTGACGGACAT			
KRT8	*forward*	GCTGACCGACGAGATCAACT	97 bp	60°C	1.69
	reverse	CATGGACAGCACCACAGATG			
KRT18	*forward*	ACAGTCTGCTGAGGTTGGAGCT	111 bp	62°C	1.71
	reverse	TCCAAGCTGGCCTTCAGATTTC			
CYP3A4	*forward*	CCTTACACATACACACCCTTTGGAAGT	382 bp	62°C	1.71
	reverse	AGCTCAATGCATGTACAGAATCCCCGGTTA			
CYP2C9	*forward*	ACGGATTTGTGTGGGAGAAGCCC	287 bp	62°C	1.67
	reverse	TGAGATGACAGGTGAGAAAAGGCA			
CYP2B6	*forward*	GGGGCACTGAAAAAGACTGA	118 bp	58°C	2.09
	reverse	AGTTCTGGAGGATGGTGGTG			
CYP1A2	*forward*	ATGGCATTGTCCCAGTCTGTT	135 bp	60°C	1.97
	reverse	TGGCTCTGGTGGACTTTTCAG			
ABCG2	*forward*	CAGGTGGAGGCAAATCTTCGT	247 bp	58°C	1.87
	reverse	ACCCTGTTAATCCGTTCGTTTT			
Albumin	*forward*	CTTGAATGTGCTGATGACAGG	157 bp	57°C	1.74
	reverse	GCAAGTCAGCAGGCATCTCAT			
α-1-AT	*forward*	CCGAAGAGGCCAAGAAACAGA	147 bp	60°C	1.79
	reverse	CAAAGGGTCTCTCCCATTTGC			
HPRT1	*forward*	GTTATGGCGACCCGCAG	107 bp	55°C	1.73
	reverse	ACCCTTTCCAAATCCTCAG			
MDH1	*forward*	GTCACGACTGTGCAGCAGCGT	117 bp	66°C	2.30
	reverse	TGGGGTTCCAAACCAGATGTCCCTG			

PSMB6	*forward*	CGGGAAGACCTGATGGCGGGA	124 bp	66°C	1.97
	reverse	TCCCGGAGCCTCCAATGGCAAA			
TBP	*forward*	CGGTTTGCTGCGGTAATCAT	122 bp	59°C	2.13
	reverse	TTTCTTGCTGCCAGTCTGGAC			
rRNA 18S	*forward*	GACTCAACACGGGAAACCTCACC	123 bp	63°C	1.81
	reverse	ACCAGACAAATCGCTCCACCAACT			
RPLP0	*forward*	TTCATTGTGGGAGCAGAC	156 bp	55°C	1.83
	reverse	CAGCAGTTTCTCCAGAGC			
**Reference Gene**	**Direction**	**Primer Sequence (5'->3')**	**Amplicon Size**	**$T_{annealing}$**	**E_{ref}**
GAPDH	*forward*	AAGGTGAAGGTCGGAGTCAA	108 bp	55°C	1.75
	reverse	AATGAAGGGGTCATTGATGG			

Notes. '$T_{annealing}$' is the annealing temperature used for each primer pair. 'E_{target}' and 'E_{ref}' are the qPCR efficiencies of target and reference gene transcripts, respectively, obtained using the dilution model and calculated by $E = 10^{(-1/slope)}$, where "slope" is the linear regression slope of a standard curve [1,2].

SUPPLEMENTARY FIGURES

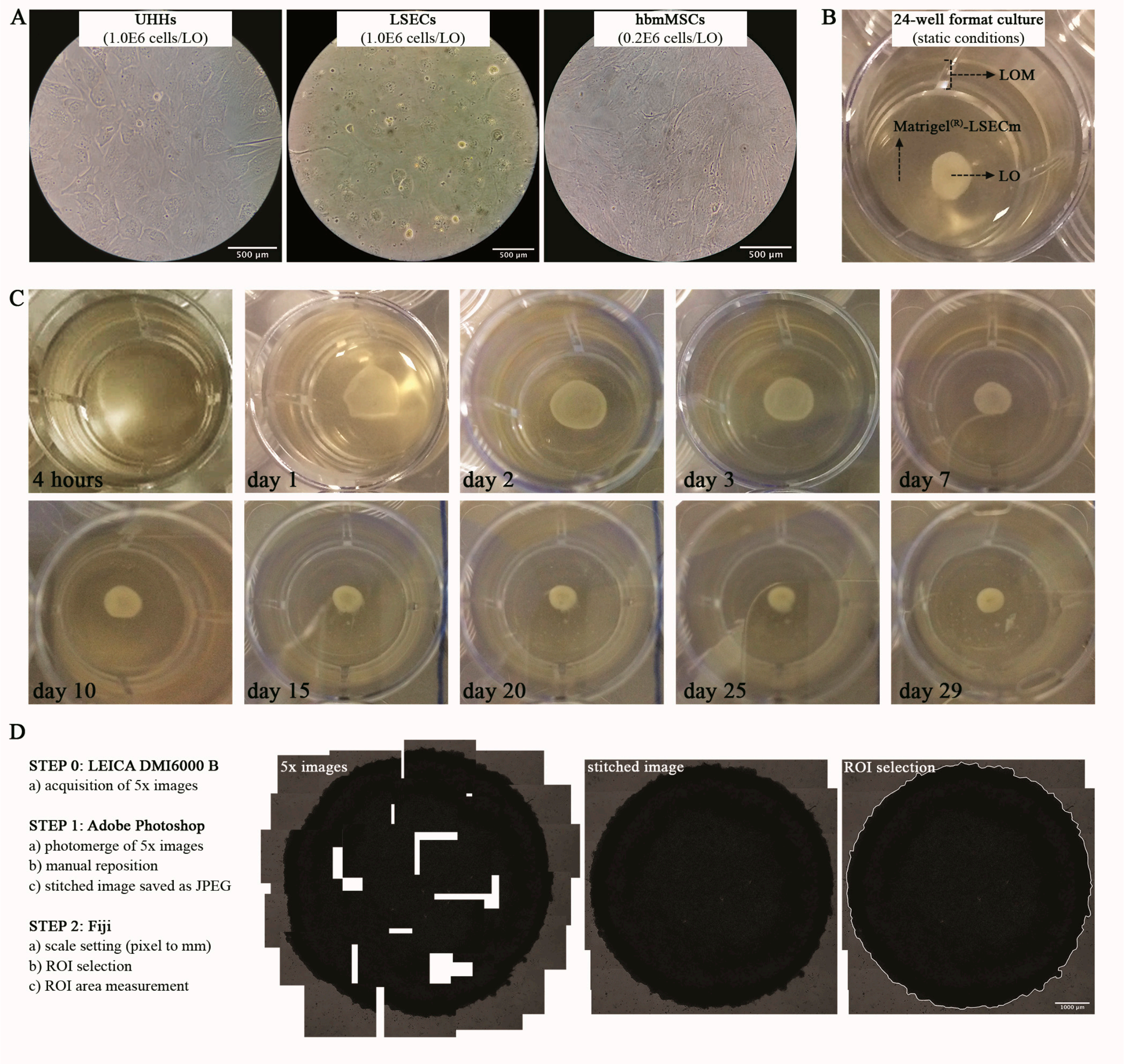

Figure S1. (**A**) Representative bright-field images by optical microscopy of cell morphology of UHHs, LSECs, and hbmMSCs mixed at the ratio of 1:1:0.2, respectively, to generate LOs (scale bar = 500 µm). (**B**) Representative morphology of LOs. Each LO is cultured with LOM in Matrigel®-coated 24-well plates, under static conditions. "LOM" refers to the liver-organoid medium. (**C**) Photographs of LOs cultured at different time intervals ranging from 4 hours to day 29. (**D**) Schematic used for measuring the 2D projection area of LOs at defined time intervals by Adobe Photoshop and Fiji (for technical details, see Materials and Methods).

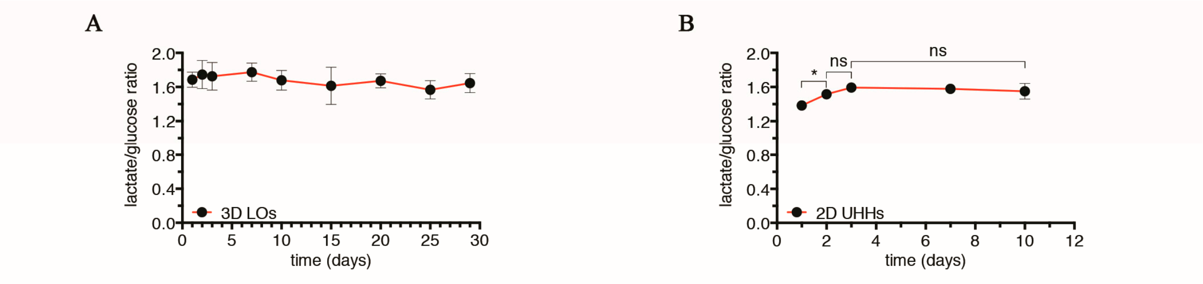

Figure S2. (**A**) Lactate to glucose molar ratio relative to LOs at defined time intervals (day 1, 2, 3, 7, 10, 15, 20, 25, and 29). For each time point, results represent means ± SD of eight independent LOs. (**B**) Lactate to glucose molar ratio relative to UHHs cultured in a 2D format for 10 days under conditions similar to those used for LOs (*i.e.*, 1E6 cells in LOM). For each time point, results represent means ± SD of four replicates. The symbols '*' and 'ns' refer to p = 0.0142 and p > 0.05, respectively (ordinary one-way ANOVA).

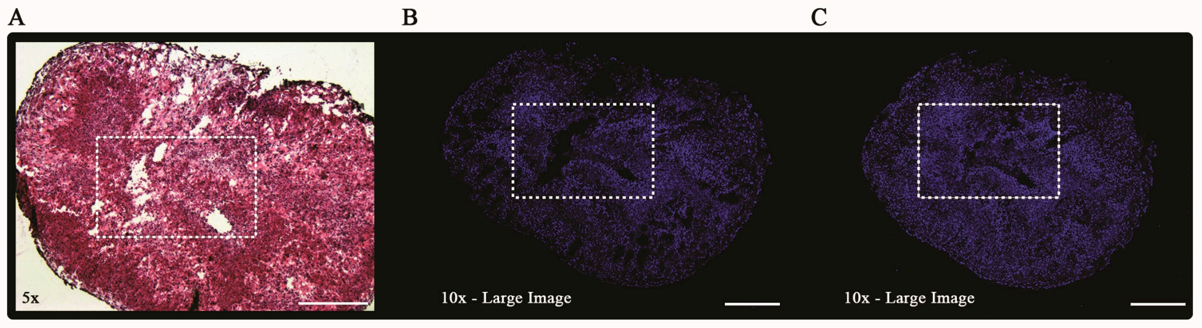

Figure S3. (**A**) H&E staining performed on frozen sections of a representative LO cultured for 29 days (5x; scale bar = 750 µm). The highlighted area indicates presumably necrotic tissue. (**B-C**) Immunofluorescence staining for the detection of nuclei (stained blue by Hoechst 33342) in different frozen sections of the same LO stained by H&E. Large images created combining more 10x acquisitions automatically are reported (scale bars = 500 µm). The highlighted areas indicate presumably necrotic tissue across sections.

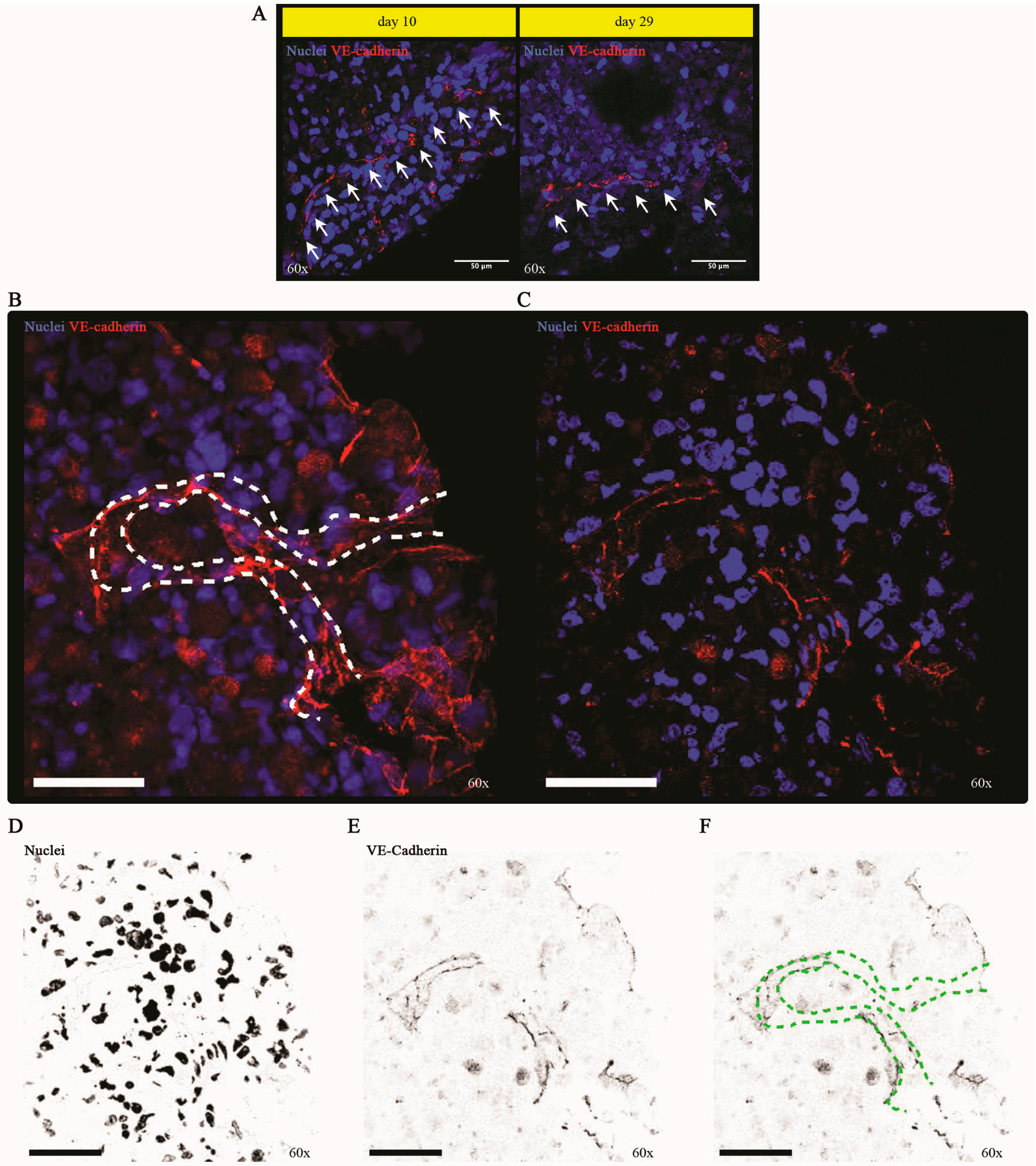

Figure S4. (**A**) Immunofluorescence staining for the detection of VE-cadherin (red) in frozen sections of LOs cultured for 10 (left panel) or 29 days (right panel). Representative images (60x; scale bars = 50 µm) are reported. Nuclei are stained by Hoechst 33342 (blue). White arrows point to intercellular tube-like structures. (**B**) Representative high-magnification image (60x; scale bars = 50 µm) after stack reconstruction of a portion of the organoid, acquired by confocal microscopy and stained for VE-cadherin (red) and nuclei (Hoechst 33342, blue). White dotted lines indicate the tube-like structures. (**C**) Representative high-magnification image (60x; scale bars = 50 µm) of a single plane from the image stack, showing VE-cadherin-positive linear structures. (**D-F**) Single-channel images with inverted colors relative to the image shown in panel C, displaying the presence of nuclei and VE-cadherin markers, respectively. The dotted green lines in panel F trace the tube-like structures highlighted in panel B.

Video S1. The animation presents a representative high-magnification image (60x) after stack reconstruction of a portion of the organoid, acquired using confocal microscopy. The sample is stained for VE-cadherin (red) and nuclei (Hoechst 33342, blue). The video sequence is as follows: it begins with a composite colour image showing the three-dimensional distribution of both nuclei and VE-cadherin signals. The second segment features a single-channel reconstruction with inverted colours, highlighting the cell nuclei. The final segment displays a single-channel reconstruction with inverted colours, illustrating the VE-cadherin distribution and the presence of tubular structures.

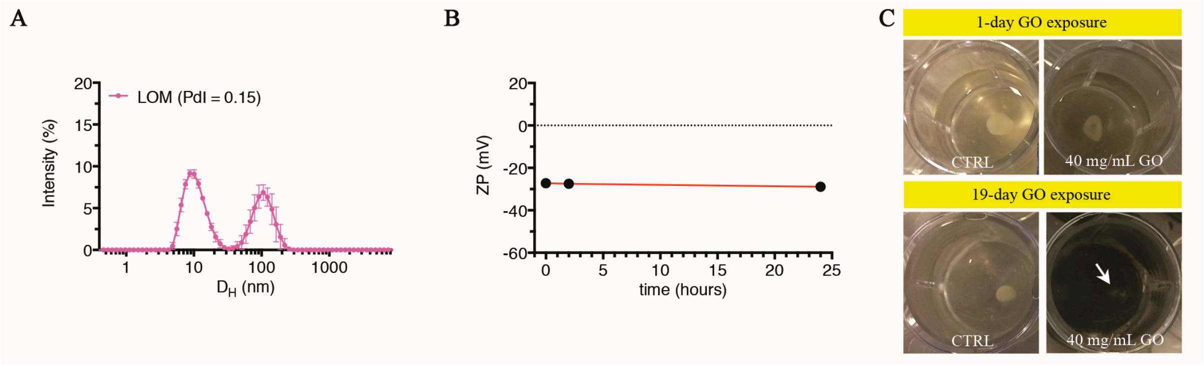

Figure S5. (**A**) Size distribution profiles of GO-free LOM, by DLS analysis. (**B**) Surface charge of particle-corona complexes after incubation of GO in LOM for 0, 2, and 24 hours by Zeta Potential (ZP) analysis. (**C**) Representative images of LOs treated with 40 µg/mL GO for 1 and 19 days or un-treated (indicated as CTRL). The white arrow points to the treated LO almost completely covered by GO deposits after 19 days of exposure.

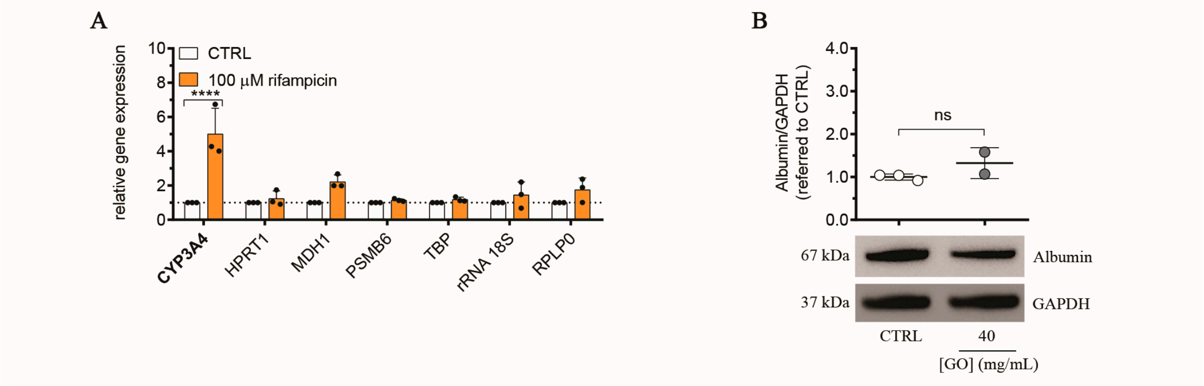

Figure S6. (**A**) Relative gene expression of CYP3A4, along with six endogenous control genes, in LOs cultured for 7-10 days and then daily treated with 100 µM rifampicin for up to 3 days or un-treated (indicated as CTRL), analysed by qPCR. Results are expressed as mean ± SD of three independent LOs per experimental condition. The symbol '****' refer to $p < 0.0001$ (two-way ANOVA). (**B**) Albumin levels in LOs daily treated with 40 µg/mL GO for up to 19 days or un-treated, analysed by western blot. Densitometric analysis of band intensities relative to two or more independent LOs per experimental condition (upper panels) and a representative blot (lower panels) are reported. GAPDH was used as internal control. Results are expressed as n-fold increase over the mean value relative to CTRL and represent means ± SD. The symbol 'ns' refers to $p > 0.05$ (unpaired Mann-Whitney U test).

RAW DATA

Western Blot

A (blot used for Figures 3C and 5B)

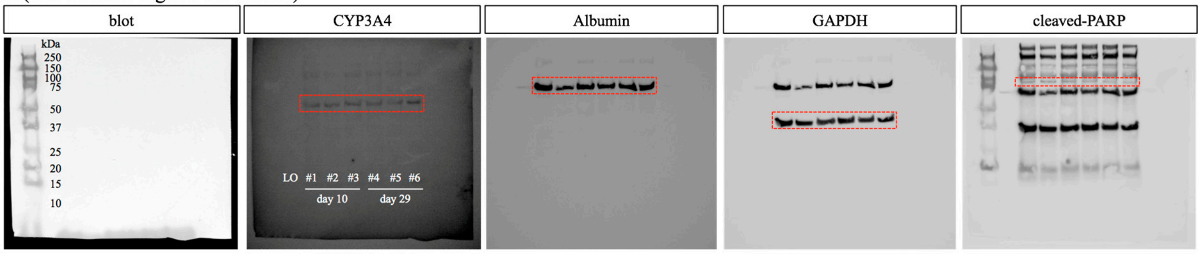

B (blot used for Figure 7C)

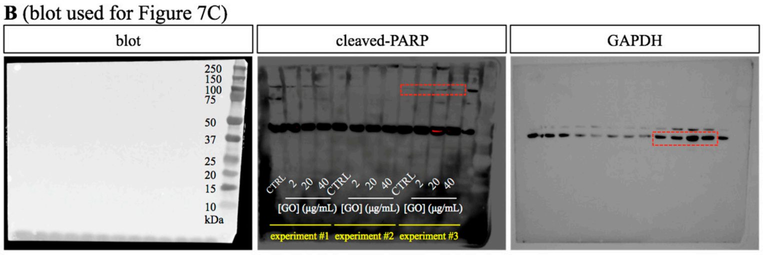

C (blot used for Figures 7E and S8B)

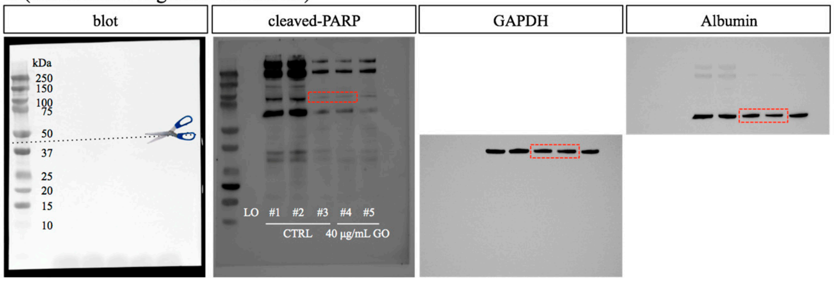

Notes. Original western blots relative to the total protein content from LOs assessed on days 10 and 29 (**A**) and LOs treated with 2-40 μg/mL GO or untreated (indicated as CTRL) for 1 (**B**) and 19 days (**C**). Samples from three independent LOs per experimental condition (except of LOs treated with 40 μg/mL GO for 19 days) were run simultaneously on the same gel, and the corresponding blot was probed consecutively with antibodies raised against CYP3A4, Albumin, cleaved-PARP or GAPDH. Red selections indicate the boundaries whereby the lanes were cropped to be reported in the main text (see **Figure 3**, panels **C**; **Figure 5** panel **B**; **Figure 7** panels **C** and **E**; **Figure S8** panel **B**).

REFERENCES

1. Romaldini, A.; Spanò, R.; Catalano, F.; Villa, F.; Poggi, A.; Sabella, S. Sub-Lethal Concentrations of Graphene Oxide Trigger Acute-Phase Response and Impairment of Phase-I Xenobiotic Metabolism in Upcyte® Hepatocytes. *Front. Bioeng. Biotechnol.* **2022**, *10*.
2. Pfaffl, M.W. A new mathematical model for relative quantification in real-time RT-PCR. *Nucleic Acids Res.* **2001**, *29*, 45e – 45.
